# Phytoconstituents and Pharmacological Activities of Indian Camphorweed (*Pluchea indica*): A Multi-Potential Medicinal Plant of Nutritional and Ethnomedicinal Importance

**DOI:** 10.3390/molecules27082383

**Published:** 2022-04-07

**Authors:** Sabrin R. M. Ibrahim, Alaa A. Bagalagel, Reem M. Diri, Ahmad O. Noor, Hussain T. Bakhsh, Gamal A. Mohamed

**Affiliations:** 1 Department of Chemistry, Preparatory Year Program, Batterjee Medical College, Jeddah 21442, Saudi Arabia; 2 Department of Pharmacognosy, Faculty of Pharmacy, Assiut University, Assiut 71526, Egypt; 3 Department of Pharmacy Practice, Faculty of Pharmacy, King Abdulaziz University, Jeddah 21589, Saudi Arabia; abagalagel@kau.edu.sa (A.A.B.); rdiri@kau.edu.sa (R.M.D.); aonoor@kau.edu.sa (A.O.N.); htbakhsh@kau.edu.sa (H.T.B.); 4 Department of Natural Products and Alternative Medicine, Faculty of Pharmacy, King Abdulaziz University, Jeddah 21589, Saudi Arabia; gahussein@kau.edu.sa

**Keywords:** *Pluchea indica*, Asteraceae, traditional uses, phytoconstituents, bioactivities, nutritional value, health and well-being

## Abstract

*Pluchea indica* (L.) Less. (Asteraceae) commonly known as Indian camphorweed, pluchea, or marsh fleabane has gained great importance in various traditional medicines for its nutritional and medicinal benefits. It is utilized to cure several illnesses such as lumbago, kidney stones, leucorrhea, inflammation, gangrenous and atonic ulcer, hemorrhoids, dysentery, eye diseases, itchy skin, acid stomach, dysuria, abdominal pain, scabies, fever, sore muscles, dysentery, diabetes, rheumatism, etc. The plant or its leaves in the form of tea are commonly used for treating diabetes and rheumatism. The plant is a rich source of calcium, vitamin C, dietary fiber, and β-carotene. Various biomolecules have been isolated from *P. indica*, including thiophenes, terpenes, quinic acids, sterols, lignans, phenolics, and flavonoids. The current review reports detailed information about the phytoconstituents and pharmacological relevance of *P. indica* and the link to its traditional uses. The reported studies validated the efficacy and safety of *P. indica*, as well as supported its traditional uses for treating various ailments and promoting health and well-being. Thus, this could encourage the development of this plant into a healthy food supplement or medicine for the prevention and treatment of various diseases. However, further studies on the drug interactions, mechanism of action, pharmacokinetics, toxicology, and metabolism, as well as clinical trials, should be carried out.

## 1. Introduction

Plants play a remarkable role in medicine development because of their capacity to biosynthesize secondary metabolites with significant bioactivities [1,2,3,4]. Traditionally, plants were utilized to treat various ailments [5,6,7]. It was reported that more than 80% of the population still rely on folk and traditional medicine, most of which are based on plant remedies according to the WHO [1]. The global herbal trade of medicinal plants has been growing exponentially at the rate of 15% annual growth and is likely to reach a scale of five trillion USD by 2050 [8]. Medicinal plants are often cheaper, more easily obtainable, and have fewer side effects than synthetic drugs [9,10,11]. Many promising compounds have been discovered from plants that can be utilized in modifying the existing drugs or designing completely new ones [1,2,3,4]. The Asteraceae (Compositae) family is one of the largest plant families, which includes about 24,000–30,000 species and 1600–1700 genera [12]. Its plants are shrubs and herbs, which have been commonly used since ancient times as herbal medicines and diets all over the world [13]. It contains well-known species, such as sunflower, chicory, coreopsis, lettuce, daisy, and dahlias, as well as several significant medicinal plants, such as chamomile, wormwood, and dandelion [14].

*Pluchea* is a flowering plants genus of the Asteraceae family, comprising about 80 species [15]. Its members are known as plucheas, camphorweeds, or fleabanes, and some are called sour bushes [16]. *Pluchea indica* (L.) Less. is an evergreen shrub found abundantly in salt marshes and mangrove swamps with a 1 to 2 m height that has an important role in maintaining the ecological balance in the coastal areas [17]. It is known as Indian camphorweed, pluchea, or marsh fleabane, Khlu (Thai), Kukrakonda, Kakronda, or Munjhu rukha (Bengali), Kuo bao ju (Chinese), luntas (Javanese), Beluntas (Malaysia, Indonesia, Bahasa), and kalapini (Philippines) [18]. This plant is mainly found in the subtropical and tropical zones of Asia, Africa, Australia, and America and in warm temperature regions of countries such as Indonesia, Malaysia, Taiwan, Australia, Mexico, and India [5,17,18]. A chemical investigation of this plant revealed the existence of various phytoconstituents: thiophenes, sesquiterpenes, quinic acids, sterols, lignans, and flavonoids. Additionally, this plant has wide folk uses and diverse bioactivities: anti-inflammatory, anti-cancer, antioxidant, anti-microbial, and insecticidal activities. The current review summarizes the reported literature on the traditional uses, phytoconstituents, and bioactivities of this plant and isolated metabolites to highlight its positive influences on human health.

## 2. Methodology

The literature search was carried out through Google Scholar, scientific databases (e.g., Scopus, PubMed, and Web of Science), and different publishers (Taylor & Francis, Elsevier, Wiley, Thieme, Bentham, Sage, Springer, and Wiley). This current work reviews the ethnomedicinal uses and phytochemical and pharmacological activities of *Pluchea indica*. This review contains 140 references dating from 1985 until January 2022.

## 3. Morphology

The plant is a branching shrub up to 2 m tall. Its leaves are light green, obovate with a sharp acute apex, serrated edge, and subtly fine trichomes decorating its lower and upper surfaces. Moreover, the leaves are alternate, shortly stalked or stalkless with membranous leaf blades [19]. The base is blunted, and leaf bones are pinnate-formed with parchment-like intervenium. An aroma is produced when the leaf blades are crushed [20,21,22]. The stem is strong, rigid, woody, and round with monopodial branches. It has taproot branches with soft-textured root fibers. The inflorescence is a capitulum that grows in dense clusters in the leaf axils and at the branch tips with violet/white corolla [19]. Its flowers have a cup-like structure that bears tiny fruits. The fruit is dry indehiscent, one-seeded, brown, and cylindrical ≈1.0 mm long [19].

## 4. Nutritional Value

The leaves are a good source of calcium, dietary fiber, and β-carotene. Sudjaroen reported the nutritional values of *P. indica*, Holy basil, and Indian mulberry leaves. It was found that *P. indica* leaves contain seven times more calcium (251 mg/100 g) and two times more β-carotene (1225 μg/100 g) than those of Holy basil leaves (32 mg/100 g and 812 μg/100 g, respectively) [23]. One hundred grams of leaves have 251 mg of calcium, whereas nearly 297 mg of calcium are found in one serving (8 oz.) of 2% fat milk [24]. One hundred grams of the leaves contains water (87.53 g), protein (1.79 g), fat (0.49 g), dietary fiber (insoluble 0.89 g, soluble 0.45 g, and total 1.34 g), carbohydrate (8.65 g), calcium (251 mg), β-carotene (1225 μg), and vitamin C (30.17 μg) [23]. *P. indica* leaves have a sweet and astringent taste and aromatic flavor [23]. Blanched or raw leaves are eaten with Nam Prik (freshly prepared chili paste) as a side dish. In addition, they are utilized as one of the ingredients in several dishes, such as Kang ped (spicy coconut milk soup) and yum (spicy and sour salad) [25].

## 5. Ethnomedicinal Uses

All parts of *P. indica* can be used medicinally, and it has a long tradition in alternative medicine for a wide variety of ailments. In Indochina, the roots’ decoction is prescribed for fever as a diaphoretic, and its leaves’ infusion is taken internally in lumbago. The leaves and roots are utilized as an astringent and antipyretic in Patna [26]. It was reported that the overconsumption of the leaves for long periods of time may cause health problems, especially for individuals suffering from cardiovascular disease and hypertension due to its high contents of chloride and sodium [27]. In Indonesia, leave infusion/decoction is utilized as an appetite stimulant, antipyretic, digestive aid, deodorant, diarrhea solution, antitussive, and emollient [28,29]. The root decoction is utilized as an astringent and antipyretic [30]. In Thailand, bark and stem decoctions are utilized to treat kidney stones and hemorrhoids, respectively, and leaves are used to cure leucorrhea, inflammation, and lumbago [31]. Leaf tea is widely consumed in Thailand as a health-promoting drink; however, drinking this tea in large amounts increases the feeling of needing to urinate due to its diuretic effect [26]. Additionally, a poultice of the fresh leaf is used for a gangrenous and atonic ulcer [32]. Arial parts are used as an ointment to treat eye diseases and itchy skin. The plant is used for treating acid stomach, dysuria, hemorrhoids, abdominal pain, stomach cramps, scabies, fever, sore muscles, dysentery, menstruation, and rheumatism [33,34,35,36]. In Malaysia, the leaves are prescribed for leucorrhea, dysentery, rheumatism, bad body odor and breath, boils, and ulcers, while roots are used to treat lumbago, fever, headache, and indigestion [37]. The chopped stem bark cigarettes are smoked to relieve sinusitis pain [38]. Indochina, the young shoots and leaves are crushed, mixed with alcohol, and applied in baths to treat scabies, as well as to the back for lumbago and to relieve rheumatic pains [38]. Currently, dried leaves and their extracts have been commercially available in Thailand as herbal tea due to their blood glucose-lowering potential [39]. In Yogyakarta, Indonesia, the leaves are used as a galactagogue to maintain, induce, and augment maternal milk production [40]. It is used orally as anti-fertility for men [20,21]. In Peninsular Malaysia, it is cultivated in gardens for its young shoots, which can be eaten raw. Its leaf decoction is used to counteract fever, and the sap expressed from leaves is used for dysentery [41]. In Dayak Pesaguan tribes, *P. indica* leaves are utilized to eliminate bad body odor, increase appetite, and overcome digestive disorders [42].

## 6. Phytochemistry

**Figure 1:**
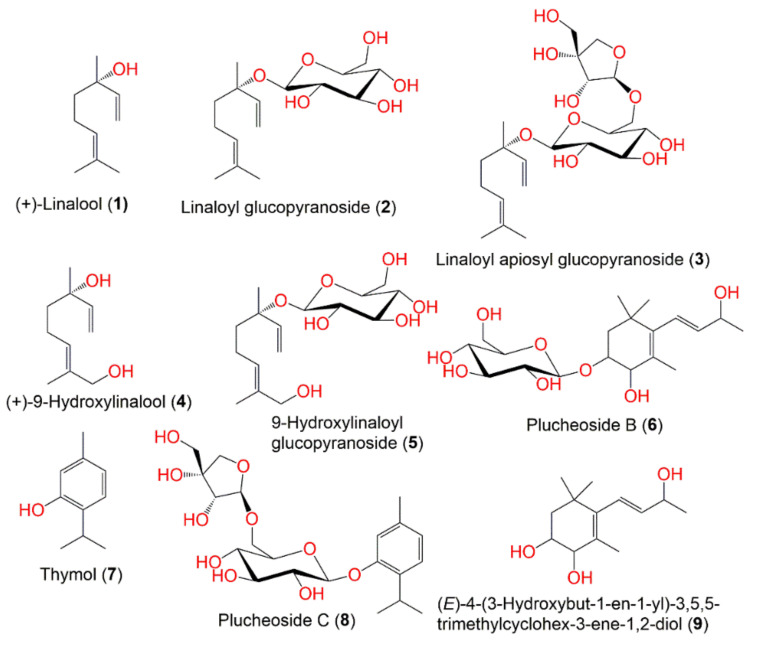
Structures of compounds **1**–**9**.

Various methods for extraction, separation, and characterization were utilized to purify and identify the chemical metabolites from various parts of *P. indica*. A total of 122 compounds have been separated and characterized. These metabolites mainly include terpenes (Figure 1), sterols, caffeoylquinic acid derivatives, flavonoids, phenolics, lignans, and thiophenes. These compounds, along with their molecular weights and formulae, plant parts and fraction/extract from which they were obtained, as well as the location of plant collection were summarized.

GC-MS analysis of the essential oil obtained from *P. indica* leaves collected from Sidoarjo and Surabaya in Indonesia using the hydro-distillation technique revealed the existence of 66 components: aldehydes, alcohols, aliphatic unsaturated hydrocarbons (cyclic, aliphatic, heterocyclic, and aromatic), ketones, esters, sulfoxides, and ethers. (10S,11S)-himachala-3-(12)-4-diene (**25**) (17.13%) was the major volatile constituent [43]. Ruan et al. investigated the chemical profiling of *P. indica* aerial parts using OC (orthogonal chromatography)-MS integrated normal-phase SiO_2_ column with reverse-phase BEH C18 column, revealing the identification of 114 metabolites: sesquiterpenes, monoterpenes, sulphated flavonoids, flavonoids, lignans, thiophenes, caffeoylquinic acid derivatives, and other phenolics. By comparing with standard references, 67 metabolites were identified, whereas 47 compounds were speculated based on MS/MS fragmentation, 10 of them were new metabolites [44]. On the other hand, the LC-MS of the leaves EtOH extract demonstrated major metabolites, including campesteryl ferulate (**33**), quercetin (**44**), 6-hydroxykaempferol 7-glucoside (**51**), 8-hydroxyluteolin 8-glucoside (**57**), apigenin 7-(2″,3″diacetylglucoside) (**54**), and trans-trismethoxy resveratrol-d4 (**97**) [45] (Table 1). It is noteworthy that *P. indica* cultivation in coastal saline land would improve its medicinal quality and increase the caffeoylquinic acid derivatives concentration [46].

## 7. Biological Activities

The traditional practices and knowledge on *P. indica* utilization rely exclusively on the observations and practical experience that are passed on verbally from one generation to the next with little documented evidence. Therefore, many studies have been carried out to understand the contemporary relevance of its traditional uses based on biological evaluation (Table 2).

### 7.1. Anti-Inflammatory Activity

*Pulchea indica* was assessed for anti-inflammatory activity using various models. The plant showed promising potential, which supported the pharmacological basis of its uses as a traditional herbal medicine for treating inflammation. *P. indica* roots’ MeOH fraction of the defatted chloroform extract was assessed for its anti-inflammatory capacity utilizing several inflammation models. The extract possessed remarkable inhibitory potential towards histamine-, carrageenin-, serotonin-, sodium urate, and hyaluronidase-induced pedal inflammation. It prohibited cotton pellet- and carrageenin-induced granuloma formation, adjuvant-induced polyarthritis, and turpentine-produced joint edema. Further, it prohibited leucocyte migration and protein exudation. This revealed the extract’s effectiveness in the chronic, proliferative, and exudative stages of inflammation [36]. Sen et al. reported that the root MeOH fraction showed significant anti-inflammatory capacity towards PAF (platelet activation factor), compound 48/80, and AA (arachidonic acid)-produced paw edema with noticeable suppression of spontaneous, as well as compound 48/80-induced histamine release from mast cells [33]. Additionally, it had prominent protective potential towards alcohol-, indomethacin-, and indomethacin-alcohol-caused ulceration, as evident by the significant decrease in acidity and gastric volume in pylorus ligated rats. Additionally, it exhibited a significant protective effect on the gastric mucosa [32]. It was suggested that its dual antiulcer and anti-inflammatory activity could be due to the 5-lipoxygenase inhibition pathway. Another study by Sen et al. demonstrated that the methanol extract of roots (doses 150 and 300 mg/kg) exhibited significant anti-inflammatory potential toward paw edema induced by glucose oxidase [63].

**Table 2:** Biological activities results of extracts and/or faction of *P. indica*.

**Plant Organ/Tested Extract/Fraction**	**Activity**	**Model**	**Results**		**Ref.**
			**Fraction/Extract** **(Tested Parameter, Dose)**	**Control**	
Root/MeOH fraction of the defatted chloroform extract	Anti-inflammatory	Carrageenin-induced paw edema/Charles Foster rats	0.103 mL (Paw volume, 100 mg/kg)	Phenylbutazone 0.204 mL (Paw volume)	[36]
			0.046 mL (Paw volume, 300 mg/kg)		
			75.2 (%Inhibition, 100 mg/kg)	Phenylbutazone 50.9 (%Inhibition)	[36]
			88.9 (%Inhibition, 300 mg/kg)		
		Carrageenin-induced paw edema/adrenalectomized rats	0.113 mL (Paw volume, 300 mg/kg)	Phenylbutazone 0.31 mL (Paw volume)	[36]
			80.7 (%Inhibition, 300 mg/kg)	Phenylbutazone 45.4 (%Inhibition)	[36]
		Mediator-induced edema/Histamine	0.030 mL (Paw volume, 300 mg/kg)	Control* 0.313 mL (Paw volume)	[36]
		Mediator-induced edema/Histamine	0.180 mL (Paw volume, 300 mg/kg)	Control* 0.403 mL (Paw volume)	[36]
		Mediator-induced edema/Histamine	0.030 mL (Paw volume, 300 mg/kg)	Control* 0.353 mL (Paw volume)	[36]
		Carrageenin-induced pleurisy/Charles Foster rats	46.9 mg (Rear paw weight, 100 mg/kg)	Phenylbutazone 24.7 mg (Rear paw weight)	[36]
			27.4 mg (Rear paw weight, 300 mg/kg)		[36]
		Cotton pellet-induced granuloma/Charles Foster rats	26.2 g (Granuloma weight, 100 mg/kg)	Phenylbutazone 15.4 g (Granuloma weight)	[36]
			21.8 g (Granuloma weight, 300 mg/kg)		[36]
			41.9 (%Inhibition, 100 mg/kg)	Phenylbutazone 65.7 (%Inhibition)	[36]
			51.8 (%Inhibition, 300 mg/kg)		[36]
		Carrageenin-induced granuloma/Charles Foster rats	1.22 g (Granuloma weight, 100 mg/kg)	Phenylbutazone 2.65 g (Granuloma weight)	[36]
			1.00 g (Granuloma weight, 300 mg/kg)		[36]
			63.1 (%Inhibition, 100 mg/kg)	Phenylbutazone 19.9 (%Inhibition)	[36]
			69.8 (%Inhibition, 100 mg/kg)		[36]
		Sodium urate-inducededema/Charles Foster rats	0.40 (Rear paw weight, 100 mg/kg)	Control* 0.68 (Rear paw weight)	[36]
			0.22 (Rear paw weight, 300 mg/kg)		[36]
Root/MeOH extract	Antiulcer	Indomethacin-induced gastric ulcer/Charles Foster rats	2.50 mm (Ulcer lesion index, 100 mg/kg)	Control* 4.7 1 (Ulcer lesion index)	[32]
			0.83 (Ulcer lesion index, 300 mg/kg)		[32]
			46.92 (%Inhibition, 100 mg/kg)		[32]
			82.37 (%Inhibition, 300 mg/kg)		
		Alcohol-induced gastric ulcer/Charles Foster rats	1.33 (Ulcer lesion index, 100 mg/kg)	Control* 4.1 (Ulcer lesion index)	[32]
			1.16 (Ulcer lesion index, 300 mg/kg)		[32]
			67.56 (%Inhibition, 100 mg/kg)		[32]
			71.70 (%Inhibition, 300 mg/kg)		[32]
		Alcohol-indomethacin-induced gastric ulcer/Charles Foster rats	5.72 (Ulcer lesion index, 100 mg/kg)	Control* 19.73 (Ulcer lesion index)	[32]
			4.36 (Ulcer lesion index, 300 mg/kg)		[32]
			71.0 (%Inhibition, 100 mg/kg)		[32]
			78.0 (%Inhibition, 300 mg/kg)		[32]
		Gastric secretion following pyloric ligation/Charles Foster rats	13.0 mEq acid (HCl)/1/h (Total acidity, 100 mg/kg)	*Control 16.25 mEq acid (HCl)/1/h	[32]
			7.5 mEq acid (HCl)/1/h (Total acidity, 300 mg/kg)		[32]
			9.00 mEq acid (HCl)/1/h (Total acidity, 100 mg/kg)	*Control 9.5 mEq acid (HCl)/1/h	[32]
			5.16 mEq acid (HCl)/1/h (Total acidity, 300 mg/kg)		[32]
			10.3 mg Tyrosin/mL (Peptic activity, 100 mg/kg)	Control* 11.03 Tyrosin/mL (Peptic activity)	[32]
			9.6 Tyrosin/mL (Peptic activity, 300 mg/kg)		[32]
		PAF-induced gastric ulcer/Charles Foster rats	15.0 mm (Ulcer lesion index, 100 mg/kg)	BW 755C 8.33 mm (Ulcer lesion index)	[64]
			11.66 mm (Ulcer lesion index, 300 mg/kg)	BW 755C 8.33 mm (Ulcer lesion index)	[64]
			35.6 (%Inhibition, 100 mg/kg)	BW 755C 64.2 (%Inhibition)	[64]
			50.2 (%Inhibition, 300 mg/kg)	BW 755C 64.2 (%Inhibition)	[64]
		PAF-induced hematological change/Charles Foster rats	41.2 (Haematocrit % change, 300 mg/kg)	BW 755C 37.6 (Haematocrit% change)	[64]
			20.4 (RBC % change)	BW 755C 14.3 (RBC % change)	[64]
			25.2 (Hemoglobin % change)	BW 755C 21.2 (Hemoglobin %change)	[64]
			15.8 (WBC % change)	BW 755C 16.6 (WBC % change)	[64]
		Croton oil-induced ear edema/Swiss A mice	3.3 mg (Weight of 5 mm ear-punch, 100 mg/kg)	*Control 19.6 mg (Weight of 5 mm ear-punch	[65]
			6.8 mg (Weight of 5 mm ear-punch, 300 mg/kg)	-	[65]
			7.2 mg (Weight of 5 mm ear-punch, 250 µg/ear)	-	[65]
			15.4 mg (Weight of 5 mm ear-punch, 500 µg/ear)	-	[65]
Root/EtOH extract	Antioxidant	Hydroxyl (OH) radical-scavenging	10.77 µg/mL (OH radicals IC_50_)	Vitamin E 33.2 µg/mL (OH radicals, IC_50_)	[63]
		CCl_4_-induced lipid peroxidation/Swiss albino mice	54.5 (%Inhibition, 300 µg/mL)	vitamin E 46.28 (%Inhibition)	[63]
		Hydrogen peroxide (H_2_O_2_)-scavenging/Charles Foster rats	65.2 (% Lysis of erythrocytes, 10 µg/mL)	BW 755C 86.9 (% Lysis of erythrocytes) Phenidone 76.8 (% Lysis of erythrocytes)	[63]
Root/EtOH extract	Cytotoxic	NPC-TW 01 cells/WST-1 colorimetric assay	14.07 (%Migration, 80 µg/mL)	-	[66]
			5.65 (%Relative migration rate, 80 µg/mL)	-	[66]
			24.57 (%Colony forming efficiency, 50 µg/mL)	-	[66]
			108.5 µg/mL (24 h) (Growth inhibition 50%)	-	[66]
		NPC-TW04/WST-1 colorimetric assay	7.76 (%Migration, 60 µg/mL)	-	[66]
			93.2 (24 h) (Growth inhibition 50%)	-	[66]
			3.47 (%Relative migration rate, 60 µg/mL)	-	[66]
Leaves/EtOAc fraction	Anti-inflammatory	EPP-induced ear edema/Male Sprague Dawley rats	8.30 µm (15 min) (ED, 3 mg/ear)	PB 3.3 µm (15 min)	[67]
			18.30 µm (30 min) (ED, 3 mg/ear)	PB 25.0 µm (30 min)	
			45 µm (60 min) (ED, 3 mg/ear)	PB 43.3 µm (60 min)	
			65 µm (120 min) (ED, 3 mg/ear)	PB 48.3 µm (120 min)	
			93.24 (15 min) (%Inhibition)	PB 97.3 (15 min) (%Inhibition)	[67]
			89 (30 min) (%Inhibition)	PB 85 (30 min) (%Inhibition)	
			73 (60 min) (%Inhibition)	PB 74 (60 min) (%Inhibition)	
			54.65 (120 min) (%Inhibition)	PB 66.28 (120 min) (%Inhibition)	
		Carrageenan-induced hind paw edema/Male Sprague Dawley rats	0.12 mL (1 h) (EV, 600 mg/kg)	DC 0.16 (1 h) (EV)	[67]
			0.20 mL (3 h) (EV, 600 mg/kg)	DC 0.21 (3 h) (EV)	
			66.2 (1 h) (%Inhibition, 600 mg/kg)	DC 55.09 (1 h) (%Inhibition)	[67]
			56.74 (3 h) (%Inhibition, 600 mg/kg)	DC 54.61 (3 h) (%Inhibition)	
Leaves/EtOH extract	Anti-inflammatory	Carrageenan-induced hind paw edema/Male Sprague Dawley rats	5.75 (180 min) (%Oedema, 300 mg/kg)	Indo 3.46 (180 min) (%Oedema)	[37]
			87.9 (%Inhibition, 300 mg/kg)	92.7 Indo (180 min) (%Inhibition)	[37]
	Analgesic	Acetic acid-induced writhing/Adult Balb/calbino mice	20.3 (15 min) (Mean of writhings)	19.0 (15 min) (Mean of writhings)	[37]
			60.7 (15 min) (%Inhibition)	63.2 (15 min) (%Inhibition)	[37]
	Cytotoxicity	EA.hy926 cells/MTT assay	54.9 (%Cell viability, 100 µg/mL)	-	[68]
			27.5 (%Cell viability, 200 µg/mL)	-	[68]
			26.4 (%Cell viability, 400 µg/mL)	-	[68]
	Lipase inhibitory	3T3-L1 adipocytes/Pancreatic lipase assay	1708.35 µg/mL (IC_50_, 250–1000 µg/mL)	Orlistat 68.23 µg/mL (IC_50_)	[69]
	Antihyperlipidemic	3T3-L1 adipocytes/Lipid accumulation	76.87 (%Inhibition, 750 µg/mL)	-	[69]
			71.93 (%Inhibition, 1000 µg/mL)	-	[69]
Leaves/H_2_O extract	Anti-dyslipidemia	Prediabetic patients/Clinical trials	109.22 mg/dL (TG, 1.5 g/once/12 weeks)	Placebo 145.56 mg/dL (TG)	[70]
			122.20 mg/dL (LDL-C, 1.5 g/once/12 weeks)	Placebo 142.07 mg/dL (LDL-C)	[70]
			57.56 mg/dL (HDL-C, 1.5 g/once/12 weeks)	Placebo 46.44 mg/dL (HDL-C)	[70]
	Fibroblast hyperproliferation inhibition	Flowcytometry analysis/ Fibroblast cultures	1.3 (%Fibroblast density, 20 µmol/L)	Control 1.5 (%Fibroblast density)	[5]
			1.0 (%Fibroblast density, 40 µmol/L)	-	[5]
			0.7 (%Fibroblast density, 80 µmol/L)	-	[5]
Leaves/MeOH extract	Antidiabetic	Normoglycemic/Wistar albino rats	72.67 mg/dL (24 h) (Plasma glucose, 200 mg/kg)	Glibenclamide 73.67 mg/dL (24 h) (Plasma glucose)	[71]
			71.67 mg/dL (24 h) (Plasma glucose, 400 mg/kg)		[71]
		STZ-induced hyperglycemia/Wistar albino rats	190.0 mg/dL (24 h) (Plasma glucose, 200 mg/kg)	Glibenclamide 168.0 mg/dL (24 h) (Plasma glucose)	[71]
			172.83 mg/dL (24 h) (Plasma glucose, 400 mg/kg)	-	[71]
	Milk production	Total milk yield/lactating rats	6.82 g/L/day (23 h) (Milk yield, 750 mg/kg)	Domperidone 7.17 g/L/day (23 h) (Milk yield)	[72]
	Growth hormone promotion		1963.25 pg/µL (Serum growth hormone level, 750 mg/kg)	Domperidone 409.46 pg/µL (Serum growth hormone level)	[72]
	Weight gain	Bodyweight gain/Wistar rat dams	8.44 (%Dams body weight gain)	Domperidone 0.66 (%Dams body weight gain)	[72]
Leaves/Et OH extract	Antioxidant	DPPH scavenging/DPPH assay	96.4 µmol TE/g fw (%DPPH scavenging, 100 mg/mL)	- Quercetin 6.70 µg/mL (IC_50_, DPPH scavenging)	[28]
			24.45 µg/mL (IC_50_, DPPH scavenging)		[41]
		DPPH scavenging/DPPH assay	42.24 µg/mL (IC_50_, DPPH scavenging)	α-Tocopherol 35.57 µg/mL (IC_50_, DPPH scavenging)	[73]
		ABTS scavenging/TEAC method	3.75 µmol TE/g fw, ABTS scavenging	-	[28]
		Ferric-reducing power/FRAP assay	81.1 (µmol TE/g fw, Ferric-reducing	-	[28]
		Inhibition of lipid oxidation/TBA method	98.5 (%Inhibition)	-	[28]
		β-Carotene-linoleic scavenging activity/β-carotene bleaching method	59.8 (%Antioxidant activity)	BHA 93.5 (% Antioxidant activity)	[73]

Control*: inflamed control; Phenylbutazone: PB; Diclofenac: DC; Indomethacin: Indo; ED: edema thickness; EV: edema volume; AA: antioxidant activity.

**Figure 2:**
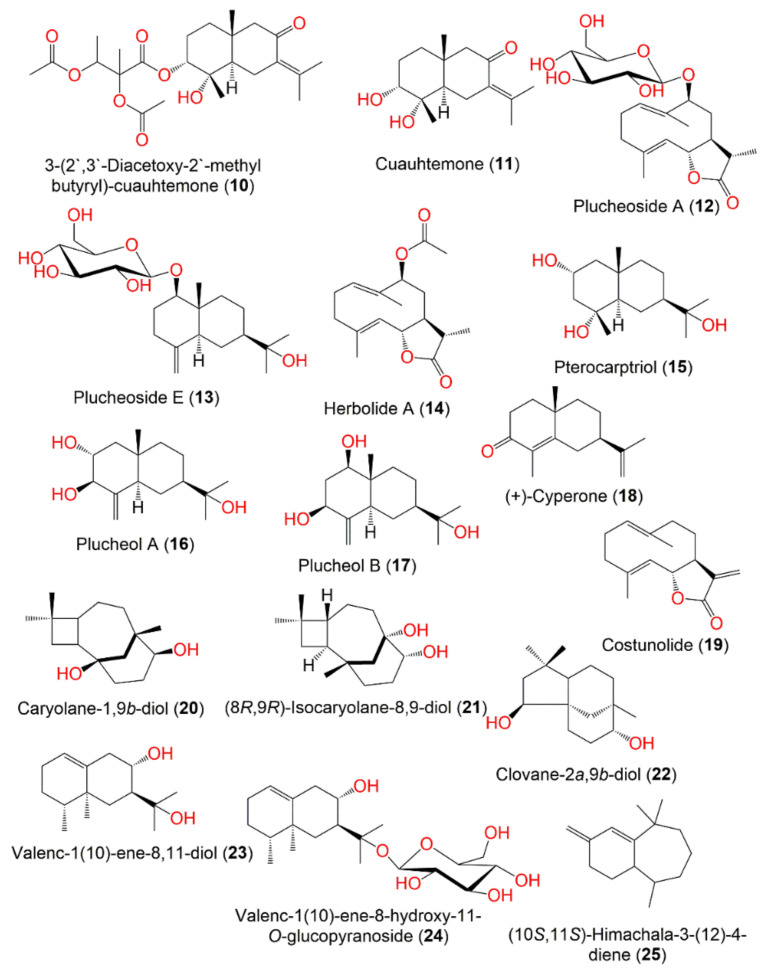
Structures of compounds **10**–**25**.

**Figure 3:**
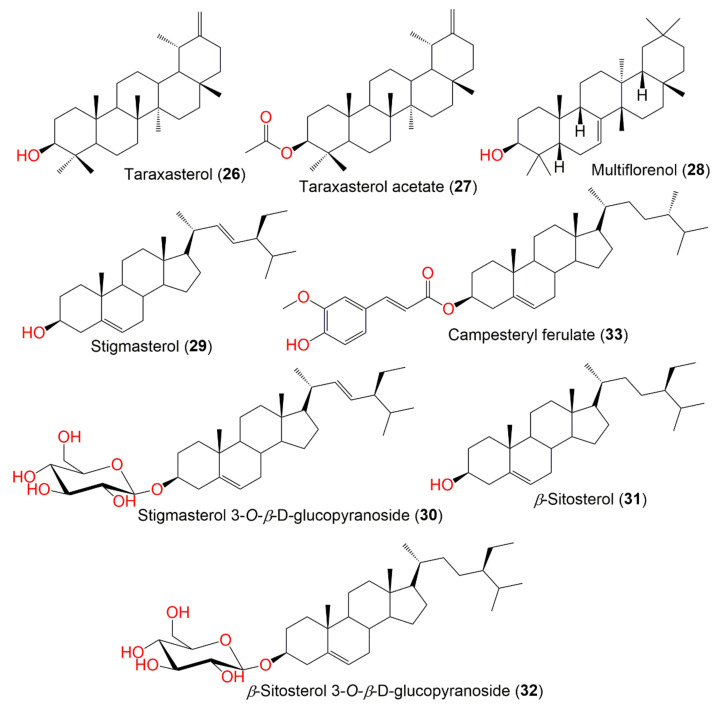
Structures of compounds **26**–**33**.

Sen et al. reported that pretreatment with root MeOH extract remarkably attenuated severe gastric mucosal vaso-congestion, hematological alteration, inflammation, necrosis, and ulceration induced by PAF administration in rats [64]. It significantly suppressed edema and decreased the hydroperoxides formation induced by croton oil in mice. It also significantly reduced lipid peroxidation induced by CC1_4_/NADPH and Fe^3+^/ADP/NADPH systems [64]. The significant anti-inflammatory activity of the EtOH extract was attributed to taraxasterol acetate that was separated from the hexane fraction (Figure 2 and Figure 3) [36].

The root MeOH extract had significant inhibitory potential on carrageenin-caused edema in rats. It remarkably prohibited granuloma formation induced by a cotton pellet in rats. It reduced the paw diameter in formaldehyde-induced arthritic rats. Additionally, it exhibited antipyretic potential on the pyrexia induced by yeast in rats [74].

The EtOAc fraction of ethanol extract of leaves exhibited potent inhibition of NO (nitric oxide) production induced by LPS (lipopolysaccharide) in RAW 264.7 macrophages and prohibited PGE2 release. It decreased iNOS (inducible nitric oxide synthase) mRNA and protein expression through suppressed iNOS promoter activity and nuclear translocation of subunit p65 of nuclear factor-kB. Moreover, it possessed anti-inflammatory potential in the inflammation acute phase, as observed in EPP (ethylphenylpropiolate)-induced ear edema and carrageenan-induced paw edema in rats. Therefore, its anti-inflammatory effectiveness was proposed to be due to iNOS suppression and NO production inhibition, which mediated via the suppression of NF-kB activation [67].

The leaf EtOH extract (dose 300 mg/kg, orally) demonstrated a remarkable inhibition of edema induced by carrageenan similar to indomethacin (dose 10 mg/kg). It noticeably prohibited (doses 10, 30, 100, and 300 mg/kg, ip) the abdominal constriction induced with acetic acid (0.6% *v*/*v*) in mice with a maximal effect at a dose of 300 mg/kg (60.7%), which was comparable to that of indomethacin (63.2%). These effects were attributed to the inhibition of PG synthesis [37]. Additionally, the EtOH extract possessed anti-inflammation potential towards TNF-α-boosted vascular inflammation on EAhy926 (human vascular endothelial) cells by reducing ROS production and lessening the ICAM-1 (intercellular adhesion molecule-1) and VCAM-1 (vascular cell adhesion molecule-1), which was partly mediated via the HO-1 up-regulation [68].

**Figure 4:**
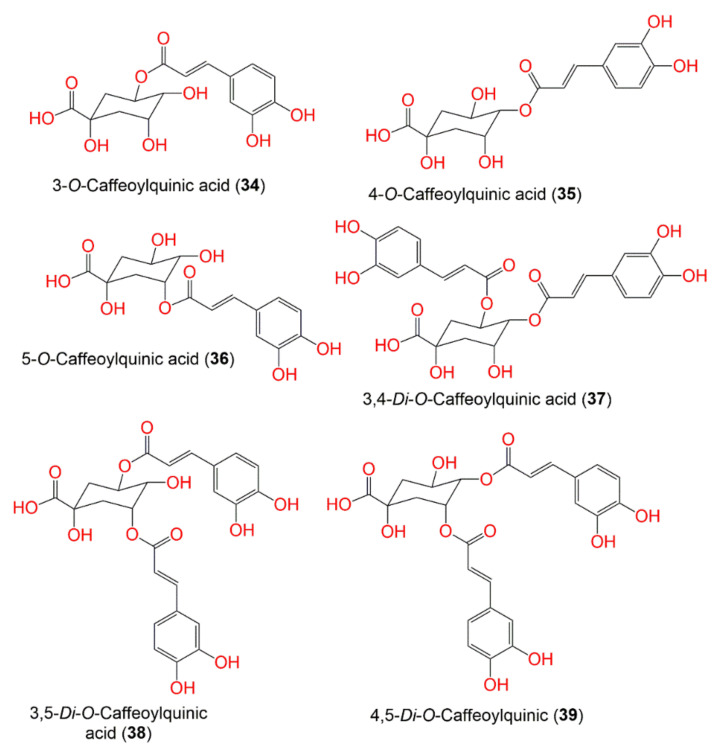
Structures of compounds **34**–**39**.

**Figure 5:**
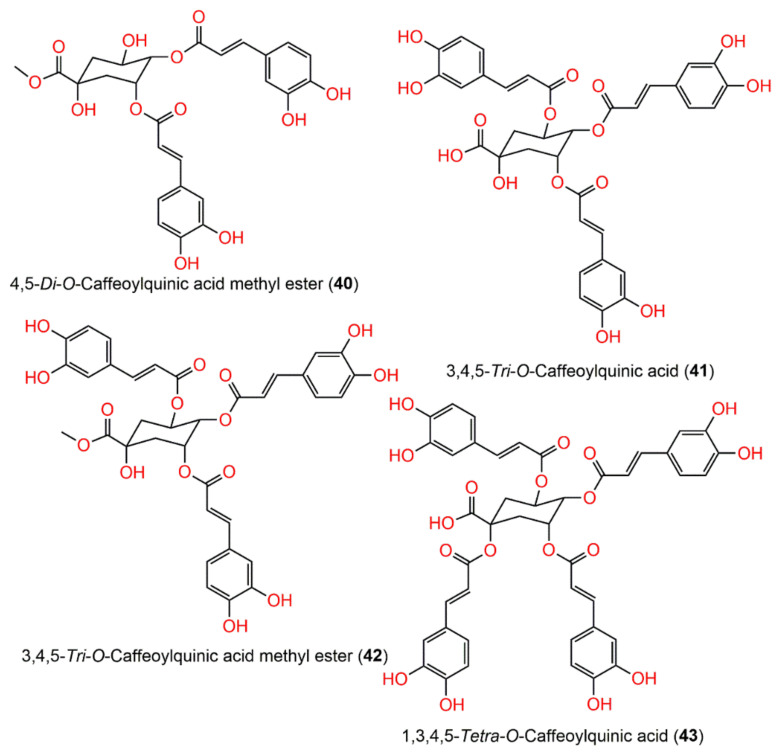
Structures of compounds **40**–**43**.

Additionally, 3,4,5-tri-*O*-caffeoylquinic acid (**41**), 1,3,4,5-tetra-O-caffeoylquinic acid (**43**), and quercetin (**44**) identified from the EtOAc fraction of the leaves were assayed for their collagenase and metalloproteinase (MMP-2 and -9) inhibitory activities in the fluorometric assay. Compounds **41** and **43** showed remarkable collagenase inhibitor potential (IC_50_ values 1.5 and 6.3 μM, respectively) compared to phosphoramidon (IC_50_ 7.4 µM); however, compound **44** had moderate potential (IC_50_ 16.9 µM). Additionally, **41** had higher MMPs’ inhibitory activities (IC_50_ 2.5 μM/MMP-2 and 6.4 μM/MMP-9) than **43** (IC_50_18.4 µM/MMP-2 and 16.8 µM/MMP-9) in comparison to chlorhexidine (IC_50_ 7.3 µM/MMP-2 and 25.2 µM/MMP-9). It was found that the number of caffeoyl groups in the quinic acid moiety and connecting position had a noticeable role in the inhibitory activity [58] (Figure 4 and Figure 5, Table 3).

New thiophenes **108**–**111**, along with the known ones **20**–**23**, **29**, **62**–**69**, **74**, **85**, **98**–**101**, **111**, **114**, **115**, **117**, **120**, and **121,** were obtained from 70% ethanol–water (EtOH) extract of *P. indica* aerial parts. Compounds **68**, **74**, **101**, **108**–**111**, **114**, **117**, and **120** possessed significant inhibitory potential towards NO production boosted by LPS in RAW 264.7 macrophages (concentration 40 µM with %inhibition ranging from 52.1 to 91.1%) compared to dexamethasone (62.2%) [51].

### 7.2. Anti-Obesity and Anti-Hyperlipidemic Activities

The incidence of dyslipidemia and obesity is currently growing at a dramatic rate throughout the world. Hyperlipidemia and obesity have resulted from energy expenditure/intake energy imbalance that leads to several health complications, including diabetes, stroke, cancer, and ischemic heart diseases [75]. The conventional anti-obesity and anti-hyperlipidemic therapy, such as orlistat and simvastatin, have severe adverse effects and limited effectiveness, often accompanied by weight gain after drug cessation [75,76]. Hence, the focus has been directed to traditional medicine as an alternative treatment for alleviating these diseases that may possess fewer undesirable influences in comparison with the conventional treatment [77].

**Figure 6:**
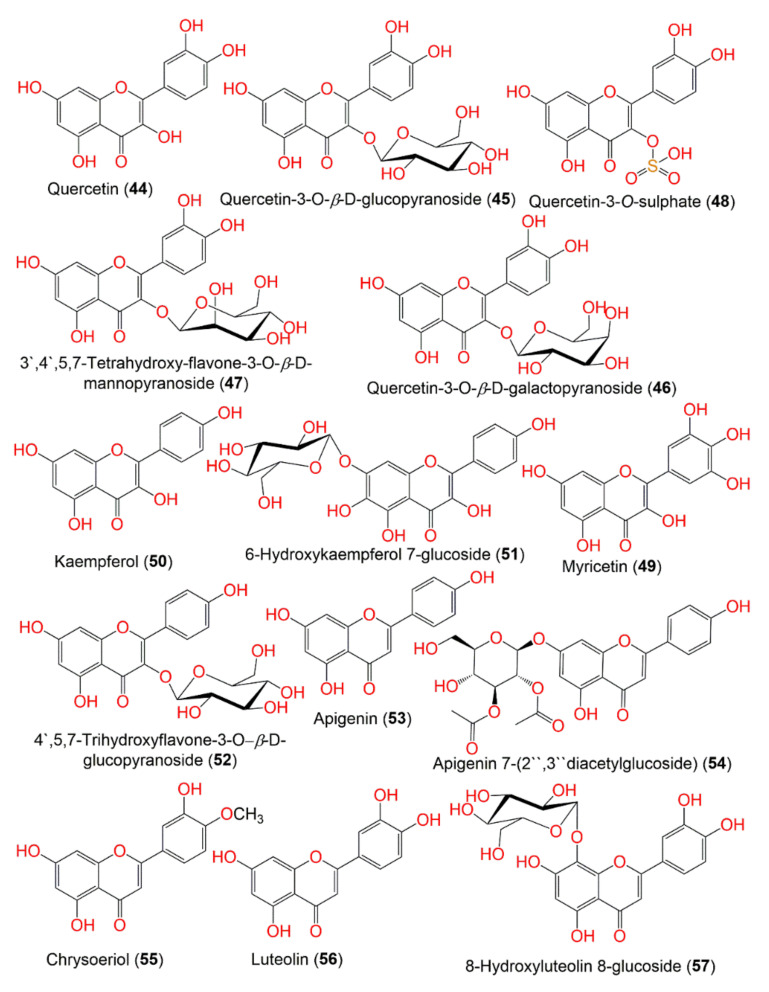
Structures of compounds **44**–**57**.

**Figure 7:**
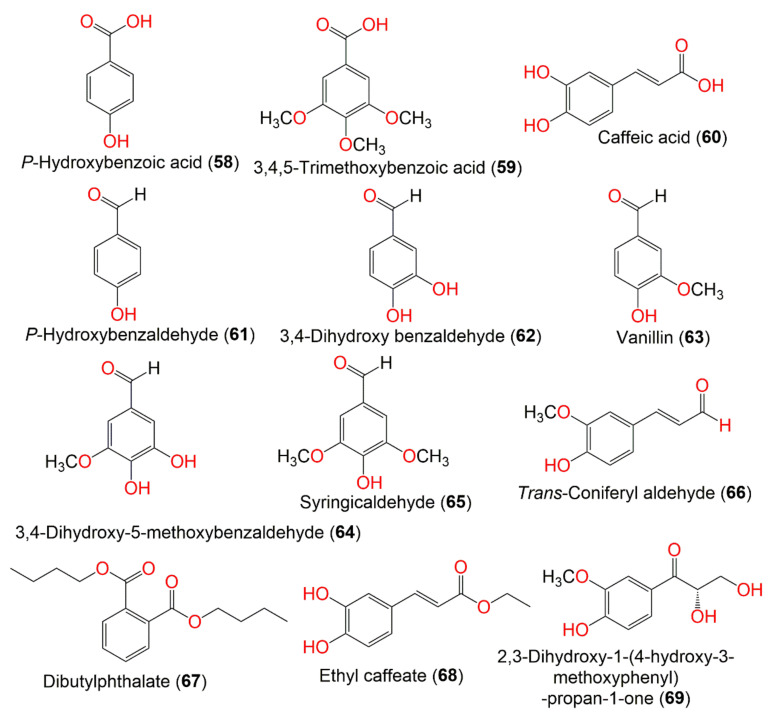
Structures of compounds **58**–**69**.

**Figure 8:**
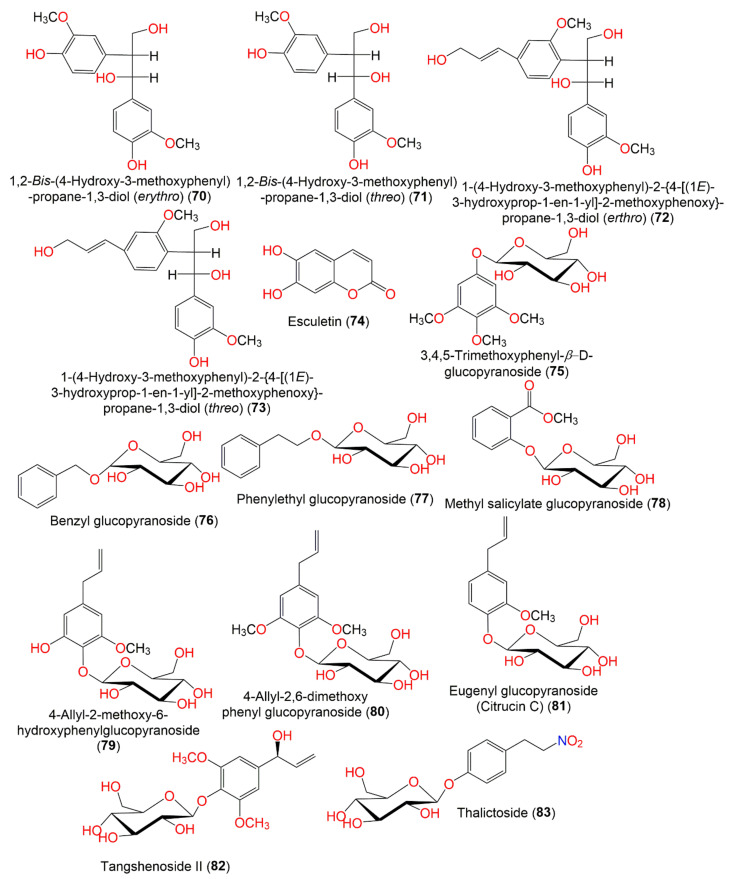
Structures of compounds **70**–**83**.

**Figure 9:**
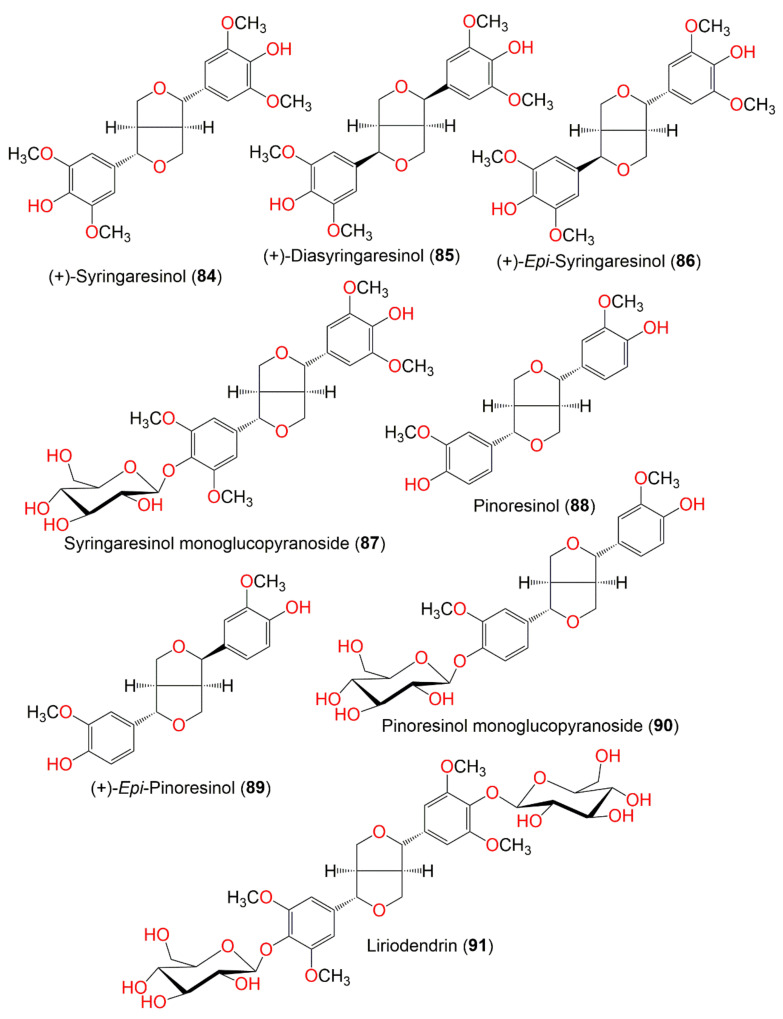
Structures of compounds **84**–**91**.

**Figure 10:**
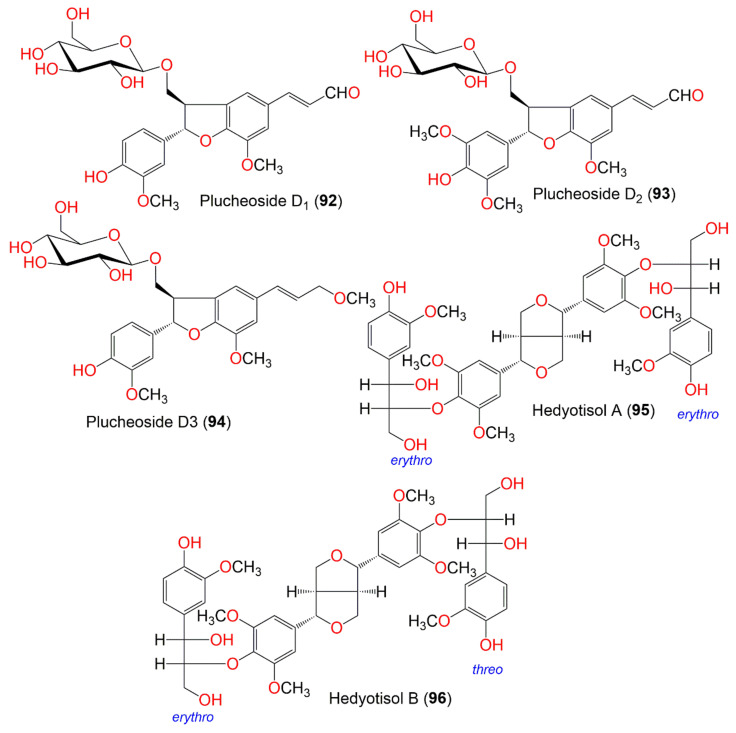
Structures of compounds **92**–**96**.

**Figure 11:**
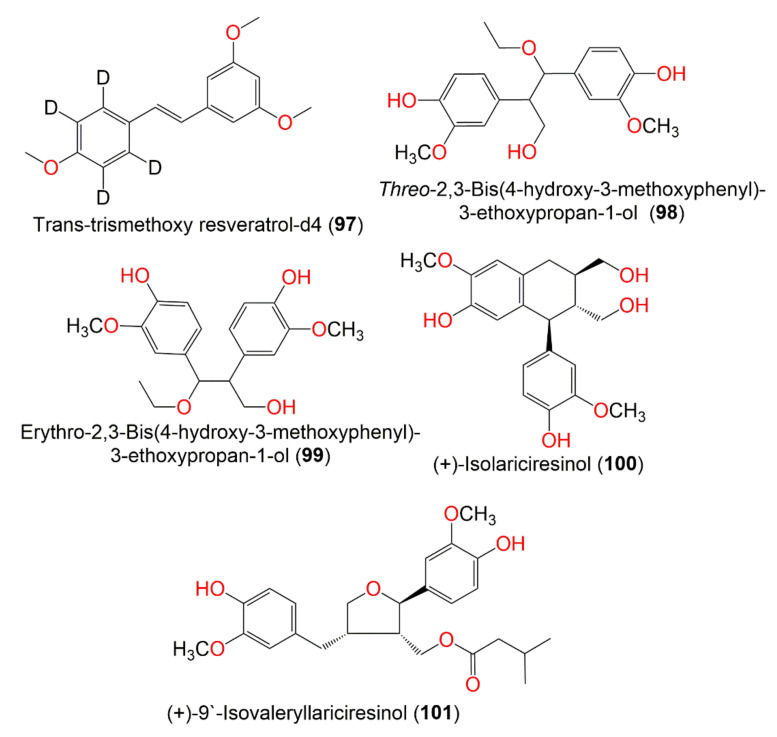
Structures of compounds **97**–**101**.

The leaf H_2_O extract (concentrations of 750 to 1000 µg/mL) markedly reduced the accumulation of lipids, suppressed adipogenesis, and modified protein, carbohydrate, glycogen, and nucleic acid concentrations in the 3T3-L1 adipocytes. Additionally, it possessed lipase inhibitory potential (concentration 250 to 1000 µg/mL) [69]. In another study, the leaf extract was found to prohibit pancreatic lipase with % inhibition ranging from 11.7% to 18.4% at a concentration ranging from 625 to 1000 ppm compared to orlistat (% inhibition from 26.6 to 36.6%) and epicatechin (% inhibition from 10.7 to 18.6%) at the same concentrations [73]. Therefore, it could be further developed into an herbal supplement for managing obesity or overweight [69]. *P. indica* tea (herb, 400 and 600 mg/kg, orally) ameliorated hyperglycemia, dyslipidemia, and obesity in high-fat diet-induced (HFD) mice. Moreover, it significantly lowered TG, TC, LDL-C, and perigonadal fat weight in HFD-treated mice; however, it increased HDL-C. This was related to its total phenolic and flavonoid contents (TFC), caffeoylquinic acid derivatives, betacaryophyllene, and gamma-gurjunene (Figure 6, Figure 7, Figure 8, Figure 9, Figure 10 and Figure 11) [70].

### 7.3. Antidiabetic Activity

Diabetes mellitus (DM) is a complicated metabolic illness that is characterized by the destruction of insulin-secreting β-cells or by insulin resistance, whereby the insulin-target organs are unresponsive to insulin action [78,79]. One of the effective strategies in diabetes management is to decrease postprandial hyperglycemia through the inhibition of α-glucosidase in the digestive system [80,81,82,83]. The available antidiabetic agents of chemical origin cause harmful side effects. Hence, developing safe and effective alternative natural antidiabetic agents is an important era of research.

Nopparat et al. reported that the pretreatment with the leaf EtOH extract (dose 100 mg/kg for 2 weeks) effectively alleviated β-cell injury produced by cytokine in STZ (streptozotocin) mice as it minimized the levels of inflammatory markers IFN-γ (interferon-γ), TNF-α (tumor necrosis factor-α), and IL-1β (interlukin1-β), along with prohibiting caspases; 3, 8, and 9, pSTAT1 phosphorylation (signal transducer and activator of transcription 1), NF-κBp65 (nuclear factor-κBp65), and iNOS. Further, it protected β-cells by boosting cell proliferation and suppressing apoptosis. The blood glucose-lowering potential of the leaf extract was attributed to the antioxidant capacities of the extract’s constituents, particularly resveratrol derivative and quercetin [45]. Additionally, it was noted that the *P. indica* leaf extracts possessed α-glucosidase and maltase inhibitory capacities that could delay the breakdown of starch into glucose and increase the glucose uptake, thereby lowering the blood glucose level [57,84].

Suriyah et al. carried out an in vitro study to assess the potential of *P. indica*’s various leaf extracts: n-hexane, CH_2_Cl_2_, EtOAc, MeOH, and H_2_O in stimulating glucose consumption in a human liver CCL-13 cell line model. It was found that *P. indica* markedly increased glucose consumption of the cells, which revealed that the antidiabetic potential of the extract was due to the stimulation of glucose uptake in the liver cells [85]. Additionally, Widyawati et al. established that the leaf H_2_O extract had higher blood glucose-reducing capability (56.37%) than glibenclamide (49.59%) and other extracts (EtOAc 19.11% and MeOH 24.27%) in rats [39]. Conversely, the leaf MeOH extract (200 and 400 mg/kg, *per os*/orally (p.o.)) was found to reduce blood glucose levels in both STZ-mediated diabetic (36.1 and 41.87%) and normal rats (35.12 and 36.1%) [71].

α-Glucosidase inhibitory assay-directed fractionation of the leaves’ EtOAc fraction yielded caffeoylquinic acid derivatives, **38** and **40**–**43,** which were isolated using SiO_2_, RP-18 CC, and HPLC and elucidated by MS and NMR analyses. Compound **42** had the highest α-glucosidase inhibitory effectiveness among the separated caffeoylquinic acid derivatives (IC_50_ 2.0 µM) compared to acarbose (IC_50_ 0.5 µM), while the other compounds displayed moderate to weak activity compared to the activity of acarbose. It was found that increasing numbers of caffeoyl groups attached to the quinic acid moiety and methyl esterification of the carboxylic group in quinic acid promoted the α-glucosidase inhibitory capacity [57].

### 7.4. Insecticidal and Herbicidal Activity

Weeds and pests that affect the plants are very harmful and represent a problem because they can prohibit the growth of cultivated plants and decrease the yield of various crops [86]. Weeds and pests can be controlled utilizing synthetic pesticides and herbicides. Nevertheless, these synthetic agents cause diverse side effects due to the toxic residual active matter that remains in the soil. Additionally, these synthetic agents can affect non-target organisms such as plants or animals. Other undesirable effects are pest resurgence and pest resistance, as well as environmental pollution [87]. The traditional methods of pest and weed control using plants or their derived metabolites are commonly used because they are safe and eco-friendly.

Yuliani and Rahayu reported that biopesticides derived from *P. indica* leaf extract caused optimal mortality (81.90% at concentration 12%) of *Spodoptera litura* larva with LC_80_ 9.88% and LC_50_ 4.00%. It also markedly prohibited the seed germination of the plant weed, *Amaranthus spinosus* [88]. Therefore, the leaf extract could be a potential bioinsecticide and bioherbicide.

### 7.5. Cytotoxicity Activity

Cancer is one of the most prevalent health problems with wide epidemiology and great mortality [89]. Its current remedies include chemotherapy, surgery, and radiation therapy [90]. These treatment strategies can result in a high recurrence rate in the case of surgical treatment, whereas chemo- and radiation therapy are lacking specificity and may harm normal cells, causing countless side effects [91].

It was found that the root EtOH extract markedly prohibited NPC (nasopharyngeal carcinoma)-TW04 and NPC-TW01 cell viability and migrations, whereas the NPC-TW04 cells had more susceptibility [92]. It noticeably increased NPC apoptosis that was triggered by up-regulation of pro-apoptotic protein Bax and suppression of anti-apoptotic protein Bcl-2 expression. Therefore, it induced the NPC cells’ apoptosis by activating p53 and regulating apoptosis-related proteins. Hence, it could be further assessed as an alternative chemotherapeutic agent for NPC [66,92]. It also prohibited K562 (human leukemia cells) proliferation, induced cell cycle arrests in the G2/M phase, and caused apoptosis [93].

The leaf and root aqueous extracts demonstrated prominent anti-migratory and anti-proliferative potential on HeLa and GBM8401 cells. They induced critical tumor suppressor molecules: phosphorylated-p53 and p21 and down-regulated phosphorylated-AKT. This effect was attributed to tannins, flavonoids, and the total phenolic contents of these extracts [91].

Moreover, the root hexane fraction exhibited a potent suppressive potential versus GBM cell growth, apparently by inducing G0/G1-phase cell cycle arrest. Further, it enhanced autophagy by increasing the formation of AVOs (acidic vesicular organelles), LC3-II expression (microtubule-associated light chain 3-II), and p38 and JNK phosphorylation [94].

**Figure 12:**
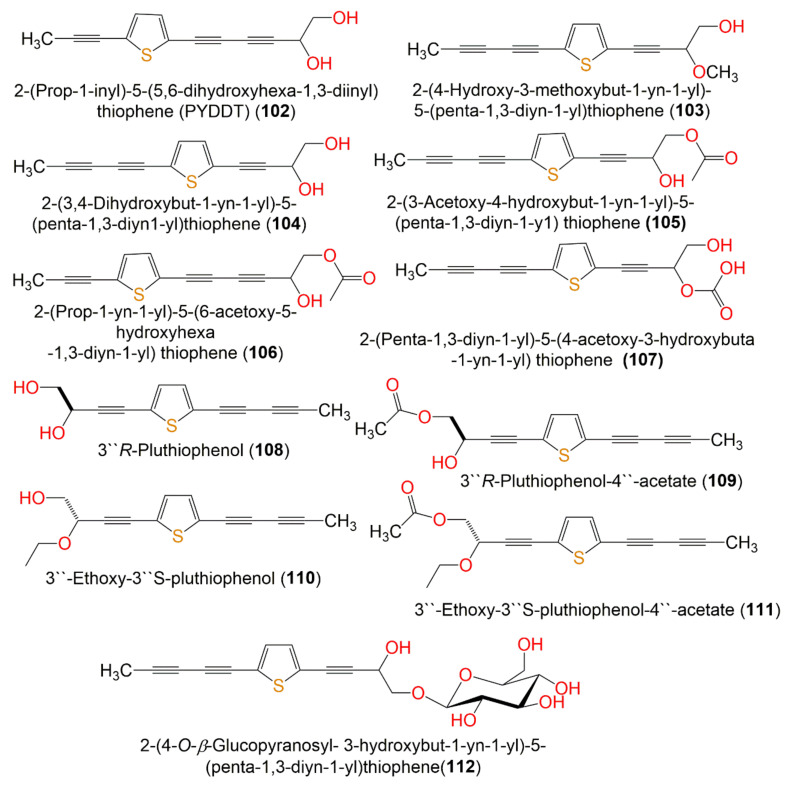
Structures of compounds **102**–**112**.

Goswami et al. prepared SLN (solid lipid nanoparticle), utilizing compound **102** purified from *P. indica*, which displayed anticancer potential on EAC (Ehrlich ascites carcinoma) cells in Swiss albino mice. It prolonged the lifespan of cancer-bearing mice and reduced tumor volume compared to PITC-2-treated groups, and it promoted the apoptosis of tumor cells [95] ((Figure 12).

**Figure 13:**
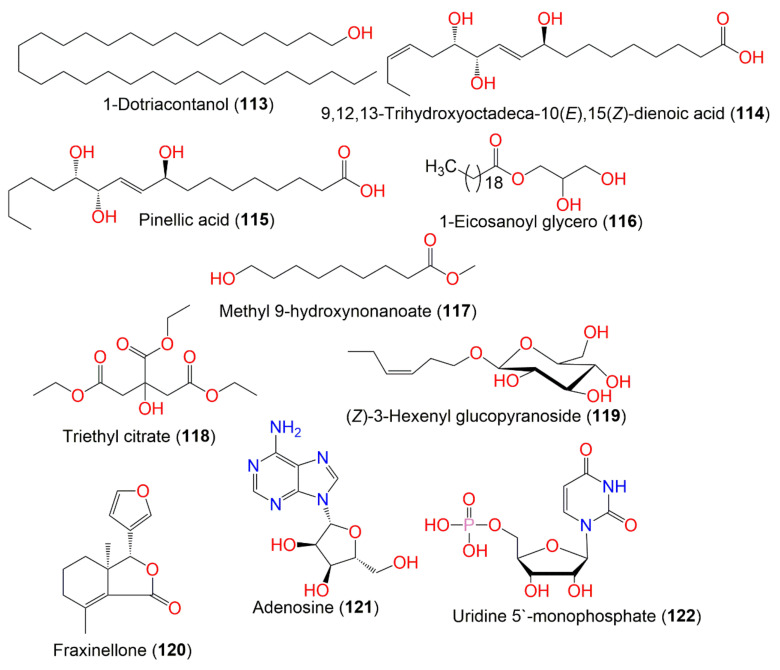
Structures of compounds **113**–**122**.

The human liver cytochrome P_450_ (CYP)-2A13 and -2A6 enzymes demonstrated a crucial function in the activation of tobacco-specific nitrosamine carcinogens and nicotine metabolism. Their prohibition could represent a strategy for smoking abstinence and decreasing the risk of lung cancer and respiratory complaints. Flavonoids **44**, **53**, **55**, **56**, **102**, **106**, and **107** isolated from the EtOAc fraction of the aerial parts by SiO_2_ CC and HPLC were evaluated for their inhibition capacity towards CYP2A6- and CYP2A13-mediated coumarin 7-hydroxylation in enzymatic reconstitution spectrofluorometric assay (Figure 12 and Figure 13). Flavonoids were the most effective metabolites at inhibiting CYP2A6 and CYP2A13 (IC_50_ ranging from 0.90 to 2.66 µM for CYP2A6 and from 0.05 to 0.80 µM for CYP2A13), followed by thiophenes (IC_50_ values 3.90 to 6.43 for CYP2A6 and 2.40 µM to 6.18 µM for CYP2A13) compared to methoxsalen (IC_50_ 0.19 and 0.43 µM, respectively). Thiophenes exhibited irreversible inhibition towards both enzymes with inactivation kinetic *K*_I_ values of 0.11–1.01 and 0.67–0.97 µM, respectively). These metabolites could have application in smoking stoppage and lessened risks of lung cancer and respiratory illnesses [59] (Figure 13).

Further, **102** purified from the EtOAc soluble fraction of the root extract of tissue cultured *P. indica* induced apoptosis and suppressed sarcoma-180 cancer cell proliferation, alongside G1 cell cycle arrest through cyclin D1, Bcl-2, and Ki-67 down-regulation, revealing its apoptotic and antiproliferative capacities versus sarcoma-180 solid tumor in vivo in mice [61].

### 7.6. Venom Neutralizing Activity

Death and injury due to snakebite are important sociomedical concerns in tropical countries [96,97]. In some hospitals, the antiserum is the only available antidote for a snakebite, which does not show complete protection against venom-induced necrosis, nephrotoxicity, or hemorrhage, and often produces adverse hypersensitivity reactions [98,99]. People in rural areas tend to choose herbal medicine from Traditional Healers because of limited or lacking hospitals [100].

*P. indica* root methanolic extract was reported to have the potential to neutralize viper venom and counter venom-induced hemorrhagic and lethality influences [97]. Further, **29** and **31** purified from the root extract were able to neutralize cobra and viper venom and antagonize cobra venom-induced cardiotoxicity, lethality, respiratory changes, and neurotoxicity, as well as potentiating the action of equine polyvalent snake venom antiserum in mice [96]. Therefore, these studies give scientific evidence for the folk use of the plant versus snakebite.

### 7.7. Hepatoprotective and Neurological Activities

Some reported findings validated the ethnomedicinal uses of *P. indica* for managing diabetic liver injury. *P. indica* leaf EtOH extract (doses 50 and 100 mg/kg for 2 weeks) ameliorated hyperglycemia-induced liver damage in STZ mice through the modulation of inflammatory responses and oxidative stress by inhibiting IL-6, TNF-α, TGF-β1, NF-κB p65, and PKC (protein kinase C), resulting in a reduction in hepatic apoptosis and improvement of hepatic architecture [101]. Further, the roots MeOH fraction displayed remarkable protective potential toward CCl_4_-induced hepatotoxicity in rats and mice. It reduced the elevated serum enzyme levels and bilirubin content. It also demonstrated a significant reduction in the prolonged pentobarbitone-produced sleeping time and plasma prothrombin time as well as a reduction in the increased bromo-sulphthalein retention produced by CCl_4_ treatment [32].

Another study reported that the root extract (doses 50–100 mg/kg, p.o.) remarkably prolonged pentobarbital sleep and reduced locomotor activity in isolated mice but not in group-housed mice. However, at high doses (dose 400 mg/kg, p.o.), it reduced locomotor activity and prolonged pentobarbital sleep in group-housed mice in comparison to diazepam (doses 0.1 and 0.5 mg/kg), which noticeably prolonged pentobarbital sleep in both isolated and group-housed mice. The effect of root extract and diazepam on pentobarbital sleep was significantly attenuated by flumazenil (1 mg/kg, i.v.). It suppressed the isolated mice’s aggressive behavior induced by social isolation. It was suggested that the GABAergic system was partly implicated in pentobarbital-caused sleep [34]. In another study, *P. indica* root extract reduced spontaneous motility, altered behavior patterns, prolonged pentobarbitone-induced sleep, and suppressed aggressive behavior patterns. Therefore, it possessed a potent CNS depressing potential [102].

### 7.8. Antifertility Activity

The population growth has raised remarkable economic and social concerns. The current strategies for controlling fertility have been debated for a long time [103]. Recently, male fertility regulation has been widely studied, aiming at developing a new male contraceptive for further inclusion of men in family planning [104]. Based on traditional methods of birth rates control, natural products represent a promising source for developing a male contraceptive. Many plants and their constituents have been investigated for their antifertility potential [105]. Interestingly, *P. indica* has been used orally in men as an antifertility alternative medicine [20,21,106].

Spermiogenesis is the last phase of spermatogenesis in which spermatids are converted to spermatozoa, which play a substantial function in the fertilization process [107]. It was reported that the leaf extract (doses 1.4, 2.8, and 5.6 mg/kg for 10 days) caused a decrease in the number of spermatid cells in male mice when the dose increased [106]. It was found that the treatment of white male adult rats with leaf tannin reduced the number of spermatozoa and spermatids [20,21]. Further, tannin remarkably reduced glutamic acid levels with an average decrease ranging from 494.43 to 341.40 mg/100 g in the semen of male white rats in comparison to the control group (547.48 mg/100 g glutamic acid level) [21,108]. Glutamic acid is needed for spermatozoa metabolism, and decreasing its level leads to the reduction in spermatozoa motility [109].

### 7.9. Wound-Healing Activity

Wound healing is a complex process that involves a variety of molecular, cellular, and biochemical reactions, which play a role in hemostasis, proliferation, inflammation, re-epithelialization, growth, and remodeling phases [110]. The healing ability is greatly influenced by systemic and local factors such as adequate nutrition, depth and location of the wound, infection, oxygenation, stress, age, obesity, diabetes, medications, and smoking [111].

In Thai traditional medicines, *P. indica* leaf was used as stringent to heal wounds and ulcers [112]. Fibroblasts represent the major type of connective tissue cells that produce an extracellular matrix accountable for maintaining tissue structural integrity [113,114]. They have a substantial function in the wound-healing proliferative phase, causing deposition of extracellular matrix [115]. Over proliferation of fibroblast during wound healing leads to the production of abnormally large amounts of collagens and extracellular matrix, resulting in scar formation and functional impairment that may further trigger permanent fibrosis [116]. The EtOH extract of leaves that was rich in flavonoids (19.44 mg/gram) exhibited remarkable antioxidant potential (IC_50_ 21.53 μg/mL) and had no effect on fibroblast 3T3 BALB C viability (IC_50_ 311.776 μg/mL) in the prestoblue cytotoxicity test. This supported the use of leaf extract as a nutrient to accelerate wound healing in the oral cavity injury [112]. Buranasukhon et al. assessed the influence of *P. indica* leaf ethanol extract and nanoparticles prepared from the leaf extract on enhancing oral wound healing using a human oral squamous carcinoma cell line. It was found that the NPs possessed wound-healing potential (concentration 62.5 µg/mL), as evident by increased cell survival and migration, as well as increased the extract’s colloidal stability in the oral spray formulation. Therefore, *P. indica* leaf extract NPs could relieve oral mucositis [113].

It was reported that the leaf extract (concentration 80 µmol/L) prevented the fibroblasts’ hyperproliferation by its major flavonoid quercetin, which prohibited collagen synthesis by interfering with the biosynthesis of procollagen (collagen precursor molecule) [5]. Increasing the synthesis of collagen is a crucial problem, particularly in the fibrotic tissue repair response due to chronic inflammation/cellular damage [117].

### 7.10. Anti-Hemorrhoidal Activity

Hemorrhoids are the major reason for rectum bleeding. They can be treated with nonsurgical or surgical treatments. Generally, initial hemorrhoid therapy is a nonsurgical treatment, which includes dietary modifications, medical management, and behavioral therapies [118].

The leaves of *P. indica* are traditionally utilized for treating hemorrhoids. This was supported by a study performed by Senvorasinh et al. They reported that the oral administration of hot *P. indica* tea H_2_O extract remarkably attenuated (dose 50 mg/kg/day) the rectal damage and hemorrhoids induced by croton oil in rats, as evident by no noticeable reduction in rectum and spleen weights. Conversely, it had no effect on gastrointestinal movement in mice, indicating it did not reduce constipation [119].

### 7.11. Antimicrobial Activity and Pharmaceutical Preparations

The leaf extract prohibited the growth of *Streptococcus mutans*, the causative organism of dental caries (MIC 10%), in the disk diffusion assay [120,121]. Further, the leaf extract toothpaste prevented dental caries in rats based on Rontgen examination results. Additionally, it reduced the virulence of mouth bacteria that initiated dental caries. Therefore, *P. indica* herbal toothpaste could treat caries in rat teeth [122]. *P. indica* leaf EtOH extract had inhibitory effects on *C. albicans* (MIC 100 mg/mL) [123]. Further, Alvionida et al. prepared different gel formulae using HPMC (hydroxypropyl methylcellulose) and carbopol 940. It was found that the gel formula with 1% carbopol 940 and 1.5% HPMC possessed antibacterial potential toward *P. aeruginosa* and *S. aureus* in the cup-plate diffusion assay [124]. Komala et al. stated that the deodorant rolls with 3 to 5% *P. indica* leaf extracts exhibited antibacterial potential towards *S. epidermidis* without causing skin irritation in both women and men. The 3% extract deodorant roll was most effective against *S. epidermidis* than the other formulae for removing the bad body odor [125], which proved its traditional use to eliminate bad odor. The leaf extract was evaluated for antibacterial potential (concentration 2.5 to 6.5%) towards *E. faecalis*, *P. gingivalis*, *F. nucleatum*, and *S. mutans*, which are accountable for root canal infections, periodontal disease, and caries. It showed significant growth inhibition towards *E. faecalis* (inhibition zone diameter (IZD) 12.6 mm), followed by *S. mutans* (IZD 12.2 mm) and *P. gingivalis* (IZD 12.2 mm) at a concentration of 6.5%, compared to chlorhexidine (IZDs 10.9, 11.4, and 10.6 mm, conc 2%) [126]. It also prohibited biofilm formation and decreased adhesion of *E. faecalis* and *F. nucleatum* in the micro-titter plate and auto-aggregation assays, respectively [127]. Hence, it could be utilized as an alternative to root canal sterilization dressing [127]. It had activity versus *S. epidermidis* (IZD 21.73 mm) and *P. aeruginosa* (IZD 21.44 mm) at 1 mg/mL [128]. Sittiwet suggested the possible urinary tract infection treatment potential of the aerial parts aqueous extract through its inhibitory effect on *E. coli* and *Klebsiella pneumoniae* [129]. Further, the root MeOH extract of *P. indica* (doses 0.5 and 1.0 mg/kg body weight) remarkably alleviated *S. typhimurium* caused typhoid fever in mice in vivo [130]. Fresh stems, roots, and twigs prohibited the growth of *S. aureus*, *P. fluorescens*, *B. cereus*, *S. typhimurium*, and *E. coli*, whereas fresh samples had more potent inhibitory potential than dried samples [131]. Farhamzah et al. prepared foot spray from the leaf EtOH extract that possessed antibacterial activity versus *B. subtilis*, which causes foot odor [132].

### 7.12. Antioxidant Activity

Oxygen radicals and other oxygen-related species are considered a cause of diverse diseases and health disorders such as degenerative and cardiovascular diseases, cancer, and immune-system and brain dysfunction [133]. The endogenous antioxidant defenses are not effective enough to alleviate or prohibit oxygen-radical-related diseases [134]. Thus, free radicals’ scavengers or exogenous antioxidants are required. The protection against free radicals or oxidizing agents can be enhanced with adequate dietary antioxidants intake.

*P. indica* fresh leaves are used in many kinds of foods, including soups, salads, and side dishes. Additionally, leaf extracts and dried leaves are commonly consumed as food supplements and tea in Thailand. Several studies confirmed the significant antioxidant potential of various extracts of *P. indica* that is highly correlated to its high total phenols and flavonoids contents. This current work summarizes these studies.

In 2002, Sen et al. reported that the MeOH fraction of *P. indica* root extract prohibited superoxide and HO^.^ radical generation, H_2_O_2_-produced lysis of erythrocytes, and CCl_4_-caused lipid peroxidation, in addition to lipoxygenase’s dioxygenase activity in the absence and presence of H_2_O_2_ [63]. Additionally, the EtOH extract of the leaves prohibited oxidation of linoleic acid and displayed ferric cyanide, ABTS, and DPPH antioxidant potential [28]. Noridayu et al. mentioned that the leaf MeOH extract had remarkable antioxidant potential (IC_50_ 24.45 µg/mL) in the DPPH assay, compared to quercetin (IC_50_ 6.70 µg/mL); however, the MeOH extract of the stem and hexane extracts of stems and leaves exhibited moderate to weak DPPH radical-scavenging capacity with IC_50_ ranging from 83.74 to 401.68 µg/mL [41]. Abdul Rahman et al. further reported the DPPH and β-carotene-linoleic scavenging potential of the *P. indica* leaf EtOH extract (IC_50_ 42.24 and 59.8 µg/mL, respectively) [73].

Interestingly, the hot water leaf extract possessed marked DPPH scavenging potential (EC_50_ 23.8 μg/mL) greater than BHT (EC_50_ 44.4 µg/mL) [135]. In 2018, Widyawati et al. evaluated the antioxidant capacity of black tea-*Pluchea* leaves drink using different proportions (0:100, 25:75, 50:50, 75:25, and 100:0% *w*/*w*). It was found that increased black tea proportion remarkably reduced iron ion-reducing power and DPPH free radical scavenging activity, except for 100% black tea proportion [136]. The leaf MeOH extracts potentially scavenged superoxide radical, reduced iron ion, and inhibited linoleic acid-β-carotene bleaching. Conversely, the EtOAc fraction exhibited a remarkable potency to scavenge superoxide radicals, reduce iron ions and TBARS, and chelate iron ions and hemoglobin. Additionally, the EtOAc fraction had an anti-warmed-over flavor (WOF) effect on duck meat, where it prevented the oxidation of linoleic acid to hexanal. Hexanal can result in a warmed-over flavor (WOF) of meat and its products [137]. Further, the antioxidant capacity of leaf extracts from mature leaves in the flowering and pre-flowering stage, as well as juvenile leaf shoots for *P. indica* samples obtained from various locations in Thailand, was estimated. Regardless of origin, the juvenile leaf shoots displayed potent antioxidant potential [84].

Sirichaiwetchakoon et al. assessed the antioxidant potential of *P. indica* aqueous tea using DPPH, ABTS, HOCl (hypochlorous acid), and peroxynitrite scavenging assays. Further, its protective potential on copper (Cu^2+^), AAPH (2,2′-azobis(2-amidinopropane) dihydrochloride), and SIN-1 (3-morpholinosydnonimine hydrochloride)-induced human LDL (low-density lipoproteins) oxidation was evaluated using TBARS assay in comparison to the known commercial green tea (CST). The tea exhibited stronger antioxidant capacity (%DPPH scavenging 51.19%, IC_50_ 245.85 μg/mL at concentrations of 75–300 μg/mL) than CST (%DPPH scavenging 41.46%, IC_50_ 315.41 μg/mL) in the DPPH, NO (nitric oxide), and peroxynitrite scavenging assays. Further, it possessed protective effects such as CST towards Cu^2+^, AAPH, and SIN-1-produced LDL oxidation that was attributed to its caffeoyl derivatives [138].

### 7.13. Other Activities

The leaf extract (dose 750 mg/kg, orally) increased growth hormone serum levels, milk production, and body weight in lactating rats [72]. The hexane extracts of stems and leaves exhibited AChE inhibitory potential that was correlated to its terpenoids content [41]. Compound **102** was purified and characterized from the EtOAc fraction of the root MeOH extract by SiO_2_ CC and spectroscopic analyses. This metabolite possessed marked anti-proliferative potential (MIC 50 µg/mL) and lytic activity on trophozoites within 4 h administration compared with metronidazole that produced complete lysis (concentration 5 µg/mL) within 2 h towards Entamoeba histolytica HM1 in the cell count assay [60].

## 8. Toxicity and Safety of *P. indica*

Many studies have been conducted to evaluate the safety of *P. indica*. The acute toxicity assessment was carried out using different doses.

Pramanik et al. proved that the MeOH extract of the leaves did not display any sign of toxicity in rats at a dose of 3200 mg/kg orally [71]. Further, the toxicity assessment of *P. indica* tea in randomized clinical trials on prediabetic humans revealed no toxicity to the liver, kidney, and blood, as evident by the lack of any remarkable variation in ALT, BUN, creatinine, CBC, and ALP of the *P. indica*-received group compared to the placebo [70]. In another study, Sirichaiwetchakoon et al. stated that *P. indica* tea (dose 600 mg/kg/day) was non-toxic to the kidney, liver, and blood in mice [139]. Nopparat et al. carried out a study to validate the toxicity of *P. indica* administration on experimental animals. The results revealed no toxic effect on kidney and liver functions, as evident by the lack of any significant variation in serum TC, TG, AST, BUN, creatinine, ALP, albumin, and ALT among all experimental groups, indicating its safety [45]. *P. indica* leaf H_2_O extract (concentration 25 to 400 µg/mL) exhibited no cytotoxic effectiveness on RAW 264.7 macrophage cells [135]. Conversely, the leaf EtOH extract did not have a cytotoxic influence (concentration 12.5–50 µg/mL) on EA.hy926 cells, while it reduced cell viability at concentrations ≥ 100 µg/mL in the MTT assay [68]. It was found that *P. indica* fresh herb H_2_O extract was safe at a concentration ≤ 1000 µg/mL on 3T3-L1 cells [69]. A sub-chronic toxicity study revealed that the oral administration of water extract was safe for mice (dose 2.6 mg/20 g b.w.); however, EtOAc and MeOH extracts (doses 1.3 and 2.6 mg/20 g b.w.) caused mortality in mice [39]. Thongpraditchote et al. reported that the root extract did not cause any mortality at a dose of up to 5 g/kg orally [34]. Additionally, it did not produce mortality in mice at a dose up to 1100 mg/kg [102].

## 9. Clinical Traits

Werdani and Widyawati stated that the regular administration of *P. indica* twice daily (2 g of tea of *P. indica* with 100 mL hot H_2_O without sugar for two months) reduced random blood glucose levels and eliminated subjective patient complaints, such as tingling in the extremities, and improved physical fatigue [140]. A randomized clinical trial in 45 prediabetic patients who received *P. indica* tea (1.5 g, daily for 12 weeks) revealed that the tea alleviated dyslipidemia and hyperglycemia, where it noticeably lowered TG and LDL-C and raised HDL-C. This supports its utilization as herbal medicine for dyslipidemia and hyperglycemia prevention [70].

## 10. Conclusion and Recommendations

**Figure 14:**
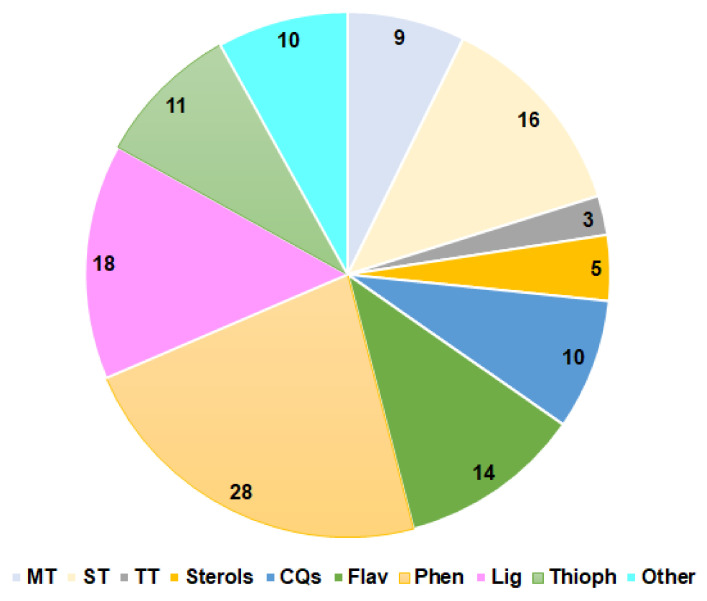
Number of metabolites from various classes reported from *P*. *indica*. MT: Monoterpenes; ST: Sesquiterpenes; TT: Triterpenes; CQs: Caffeoylquinic acid derivatives; Flav: Flavonoids; Phen: Phenolics; Lig: Lignans; Thioph: Thiophenes.

**Figure 15:**
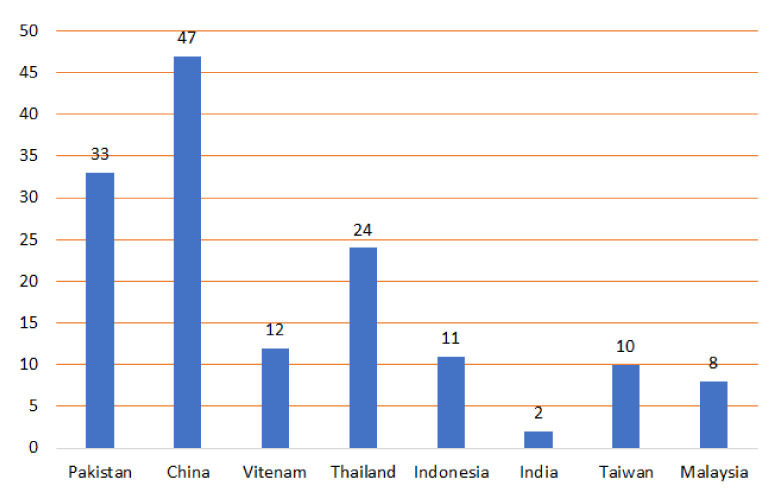
Number of reported metabolites from *P*. *indica* from various countries.

**Figure 16:**
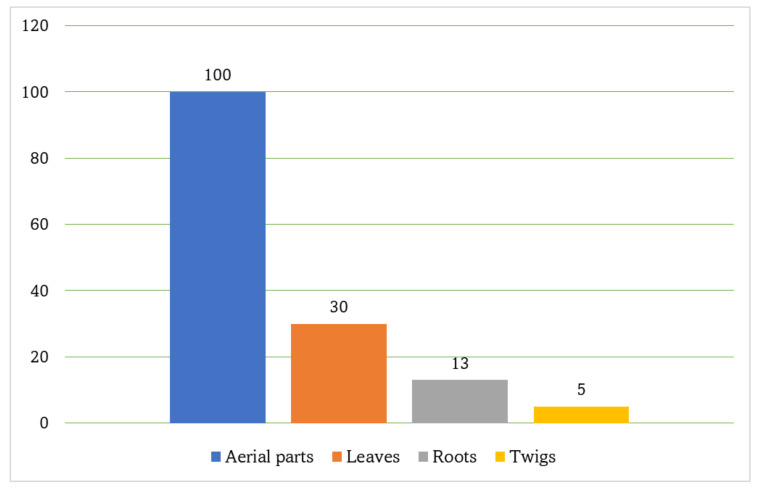
Number of metabolites isolated from different parts of *P*. *indica*.

Medicinal plants and their phytoconstituents have been proven to have a significant contribution to the prevention and therapy of several diseases and to have diverse pharmacological properties. Encouragingly, the current work reviewed several promising bioactivities that are induced by a variety of phytoconstituents of *P*. *indica*. This plant is widely used by various traditional medicines for treating various diseases, especially diabetes and its related complications. Many studies have been carried out to prove these traditional uses. The plant studied from various regions has terpenoids and caffeoylquinic acid derivatives, as well as high phenolics and flavonoid contents (TFC), which confer most of the characteristic bioactivities of this plant (Figure 14 and Figure 15). The majority has been purified from aerial parts (Figure 16).

It is noteworthy that the reported studies in this work validate the efficacy and safety of *P. indica* and support its traditional uses for treating various ailments and promoting health and well-being, particularly for people for whom modern health care is not easily accessible. Therefore, reported results could encourage the food supplement manufacturers to develop this plant into a healthy food supplement or medicine for the prevention and treatment of various diseases.

*P. indica* root and leaf extracts revealed anti-inflammatory potential towards different types of induced inflammations using various models. The suppression of NF-κB, attenuation of antioxidant enzymes, up-regulation of HO-1, and inhibition of PGE2 and 5-LOX are the reported molecular mechanisms for the anti-inflammatory potential of *P. indica*.

*P. indica* leaves/tea could have the potential to prevent and treat metabolic syndrome because they possess blood glucose and lipid-lowering effects. The antihyperlipidemic effect was due to the suppression of adipogenesis and lipase inhibition, whereas the antidiabetic effect was due to the protection of β-cell towards inflammatory responses and apoptosis, α-glucosidase and maltase inhibition, and stimulation of glucose uptake. Further, caffeoylquinic acid derivatives of the leaves were proven to have α-glucosidase inhibitory potential that was promoted by increased numbers of caffeoyl groups linked to quinic acid moiety and methyl esterification of quinic acid’s carboxylic group. The discovery of natural anticancer agents is a major concern of scientists. The reviewed plant was established to have cytotoxic potential, which is most often linked to chemoprevention. The plant was found to promote apoptosis and inhibit the migration and proliferation of diverse cancer cell lines. Further studies are warranted to specify the bioactive compound(s) accountable for the induction of these effects and to elucidate the mechanisms of *P. indica* on in vivo models.

Additionally, the venom-neutralizing potential of *P. indica* root was attributed to its phytoconstituents: stigmasterol and beta-sitosterol. The plant showed liver injury, hemorrhoids, and wound-healing capacity.

Several studies confirmed the significant antioxidant potential of various extracts of *P. indica* that is highly correlated to its high total phenols and flavonoids contents. Therefore, the plant may positively contribute to dietary antioxidant intake. Moreover, *P. indica* could be further developed into functional food and nutraceutical products.

*P. indica* extracts and their pharmaceutical preparations possessed antibacterial capacity towards the causative organisms of dental caries, bad body odor, and urinary tract infections.

Despite the diverse studies carried out on *P. indica*, a limited number of studies focus on the isolation of their chemical constituents and evaluation of their activities. Further, the clinical and toxicity studies suggested the safety of this plant for human use. Based on these facts, the authors hope that this review highlights the role of *P. indica* in various treatments and recommend that further phytochemical and clinical research should be conducted on this traditional medicinal plant for the discovery of safer drugs.

## Figures and Tables

**Table 1:** List of reported metabolites from *Pluchea indica*.

Compound Name	Plant Part	Extract/Fraction	Mol. Wt.	Mol. Formula	City, Country	Ref.
Monoterpenes						
(+)-Linalool (**1**)	Aerial parts	Polar fraction of MeOH extract	154	C_10_H_18_O	Drigh Road, Karachi, Pakistan	[47]
Linaloyl glucopyranoside (**2**)	Aerial parts	Polar fraction of MeOH extract	316	C_16_H_28_O_6_	Drigh Road, Karachi, Pakistan	[47]
Linaloyl apiosyl glucopyranoside (**3**)	Aerial parts	Polar fraction of MeOH extract	448	C_21_H_36_O_10_	Drigh Road, Karachi, Pakistan	[47]
(+)-9-Hydroxylinalool (**4**)	Aerial parts	Polar fraction of MeOH extract	170	C_10_H_18_O_2_	Drigh Road, Karachi, Pakistan	[47]
9-Hydroxylinaloyl glucopyranoside (**5**)	Aerial parts	Polar fraction of MeOH extract	332	C_16_H_28_O_7_	Drigh Road, Karachi, Pakistan	[47]
Plucheoside B (**6**)	Aerial parts	Polar fraction of MeOH extract	388	C_19_H_32_O_8_	Drigh Road, Karachi, Pakistan	[47]
Thymol (**7**)	Aerial parts	Polar fraction of MeOH extract	150	C_10_H_14_O	Drigh Road, Karachi, Pakistan	[48]
Plucheoside C (**8**)	Aerial parts	Polar fraction of MeOH extract	444	C_21_H_32_O_10_	Drigh Road, Karachi, Pakistan	[48]
(E)-4-(3-Hydroxybut-1-en-1-yl)-3,5,5-trimethylcyclohex-3-ene-1,2-diol (**9**)	Aerial parts	Polar fraction of MeOH extract	226	C_13_H_22_O_3_	Drigh Road, Karachi, Pakistan	[47]
Sesquiterpenes						
3-(2′,3′-Diacetoxy-2′-methyl butyryl)-cuauhtemone (**10**)	Leaves	CHCl_3_ fraction of EtOH extract	452	C_24_H_36_O_8_	Nakornpathom, Thailand	[49]
Cuauhtemone (**11**)	Leaves	CHCl_3_ fraction of EtOH extract	252	C_15_H_24_O_3_	Nakornpathom, Thailand	[49]
Plucheoside A (**12**)	Aerial parts	Polar fraction of MeOH extract	412	C_21_H_32_O_8_	Drigh Road, Karachi, Pakistan	[47]
Plucheoside E (**13**)	Aerial parts	Polar fraction of MeOH extract	400	C_21_H_36_O_7_	Drigh Road, Karachi, Pakistan	[48]
Herbolide A (**14**)	Aerial parts	Polar fraction of MeOH extract	292	C_17_H_24_O_4_	Drigh Road, Karachi, Pakistan	[47]
Pterocarptriol (**15**)	Aerial parts	Polar fraction of MeOH extract	256	C_15_H_28_O_3_	Drigh Road, Karachi, Pakistan	[48]
Plucheol A (**16**)	Aerial parts	Polar fraction of MeOH extract	254	C_15_H_26_O_3_	Drigh Road, Karachi, Pakistan	[48]
Plucheol B (**17**)	Aerial parts	Polar fraction of MeOH extract	254	C_15_H_26_O_3_	Drigh Road, Karachi, Pakistan	[48]
(+)-Cyperone (**18**)	Roots	CH_2_Cl_2_ fraction of MeOH extract	218	C_15_H_22_O	Kaohsiung, Taiwan	[50]
Costunolide (**19**)	Roots	CH_2_Cl_2_ fraction of MeOH extract	232	C_15_H_20_O_2_	Kaohsiung, Taiwan	[50]
Caryolane-1,9β-diol (**20**)	Aerial parts	*n*-Hexane fraction of EtOH extract	238	C_15_H_26_O_2_	Hepu, Guangxi, China	[51]
(8R,9R)-Isocaryolane-8,9-diol (**21**)	Aerial parts	*n*-Hexane fraction of EtOH extract	238	C_15_H_26_O_2_	Hepu, Guangxi, China	[51]
Clovane-2α,9β-diol (**22**)	Aerial parts	*n*-Hexane fraction of EtOH extract	238	C_15_H_26_O_2_	Hepu, Guangxi, China	[51]
Valenc-1(10)-ene-8,11-diol (**23**)	Aerial parts	*n*-Hexane fraction of EtOH extract	238	C_15_H_26_O_2_	Hepu, Guangxi, China	[51]
Valenc-1(10)-ene-8-hydroxy-11-O-glucopyranoside (**24**)	Aerial parts	EtOAc fraction of MeOH extract	400	C_21_H_37_O_7_	China	[52]
(10S,11S)-Himachala-3-(12)-4-diene (**25**)	Leaves	Essential oil	204	C_15_H_24_	Sidoarjo and Surabaya, Indonesia	[43]
Triterpenes						
Taraxasterol (**26**)	Leaves	*n*-Hexane fraction of MeOH extract	426	C_30_H_50_O	Gia Lam, Hanoi, Vietnam	[53]
Taraxasterol acetate (**27**)	Leaves	*n*-Hexane fraction of MeOH extract	468	C_32_H_52_O_2_	Gia Lam, Hanoi, Vietnam	[53]
Multiflorenol (**28**)	Aerial parts	*n*-Hexane fraction of MeOH extract	426	C_30_H_50_O	China	[52]
Sterols						
Stigmasterol (**29**)	Aerial parts	*n*-Hexane fraction of EtOH extract	412	C_29_H_48_O	Hepu, Guangxi, China	[51]
	Twigs	*n*-Hexane fraction of MeOH extract	-	-	Vietnam	[54]
	Leaves	*n*-Hexane fraction of MeOH extract	412	C_29_H_48_O	Gia Lam, Hanoi, Vietnam	[53]
Stigmasterol 3-O-β-D-glucopyranoside (**30**)	Aerial parts	Polar fraction of MeOH extract	574	C_35_H_58_O_6_	Drigh Road, Karachi, Pakistan	[47]
	Twigs	EtOAc fraction of MeOH extract	-	-	Vietnam	[54]
	Leaves	EtOAc fraction of MeOH extract	-	-	Gia Lam, Hanoi, Vietnam	[53]
β-Sitosterol (**31**)	Twigs	*n*-Hexane fraction of MeOH extract	414	C_29_H_50_O	Vietnam	[54]
β-Sitosterol 3-O-β-D-glucopyranoside (**32**)	Leaves	EtOAc fraction of MeOH extract	576	C_35_H_60_O_6_	Gia Lam, Hanoi, Vietnam	[53]
Campesteryl ferulate (**33**)	Leaves	EtOH extract	576	C_38_H_56_O_4_	Hat Yai, Songkhla, Thailand	[45]
Caffeoylquinic acid derivatives						
3-O-Caffeoylquinic acid (**34**)	Aerial parts	Acidified MeOH	354	C_16_H_18_O_9_	Seberang Perai, Malaysia	[55]
	Leaves	50% EtOH extract	-	-	Different provinces in Thailand	[56]
4-O-Caffeoylquinic acid (**35**)	Leaves	50% EtOH extract	354	C_16_H_18_O_9_	Different provinces in Thailand	[56]
5-O-Caffeoylquinic acid (**36**)	Aerial parts	Acidified MeOH	354	C_16_H_18_O_9_	Seberang Perai, Malaysia	[55]
	Leaves	50% EtOH extract	-	-	Different provinces in Thailand	[56]
3,4-Di-O-Caffeoylquinic acid (**37**)	Aerial parts	Acidified MeOH	-	-	Seberang Perai, Malaysia	[55]
	Leaves	50% EtOH extract	530	C_26_H_26_O_12_	Different provinces in Thailand	[56]
	Leaves	50% EtOH extract	-	-	Different provinces in Thailand	[46]
3,5-Di-O-Caffeoylquinic acid (**38**)	Aerial parts	Acidified MeOH	516	C_25_H_24_O_12_	Seberang Perai, Malaysia	[55]
	Leaves	EtOAc fraction of MeOH extract	-	-	Yogyakarta, Indonesia	[57]
	Leaves	50% EtOH extract	-	-	Different provinces in Thailand	[56]
	Leaves	50% EtOH extract	-	-	Different provinces in Thailand	[46]
4,5-Di-O-Caffeoylquinic acid (**39**)	Aerial parts	Acidified MeOH	516	C_25_H_24_O_12_	Seberang Perai, Malaysia	[55]
4,5-Di-O-caffeoylquinic acid methyl ester (**40**)	Leaves	EtOAc fraction of MeOH extract	530	C_26_H_26_O_12_	Yogyakarta, Indonesia	[57]
	Leaves	50% EtOH extract	-	-	Different provinces in Thailand	[56]
3,4,5-Tri-O-Caffeoylquinic acid (**41**)	Leaves	EtOAc fraction of MeOH extract	678	C_34_H_30_O_15_	Khon Kaen, Thailand	[58]
	Leaves	EtOAc fraction of MeOH extract	-	-	Yogyakarta, Indonesia	[57]
3,4,5-Tri-O-Caffeoylquinic acid methyl ester (**42**)	Leaves	EtOAc fraction of MeOH extract	692	C_35_H_32_O_15_	Yogyakarta, Indonesia	[57]
1,3,4,5-Tetra-O-Caffeoylquinic acid (**43**)	Leaves	EtOAc fraction of MeOH extract	840	C_43_H_36_O_18_	Khon Kaen, Thailand	[58]
	Leaves	EtOAc fraction of MeOH extract	-	-	Yogyakarta, Indonesia	[57]
Flavonoids						
Quercetin (**44**)	Leaves	EtOAc fraction of MeOH extract	302	C_15_H_10_O_7_	Khon Kaen, Thailand	[58]
	Aerial parts	Acidified 50% MeOH	-	-	Bogor, west Java, Indonesia	[29]
	Aerial parts	EtOAc fraction of MeOH extract	-	-	Chantaburi, Thailand	[59]
Quercetin-3-O-β-D-glucopyranoside (**45**)	Aerial parts	EtOAc fraction of MeOH extract	464	C_21_H_20_O_12_	China	[52]
	Aerial parts	Acidified MeOH	-	-	Seberang Perai, Malaysia	[55]
Quercetin-3-O-β-D-galactopyranoside (**46**)	Aerial parts	Acidified MeOH	464	C_21_H_20_O_12_	Seberang Perai, Malaysia	[55]
3′,4′,5,7-Tetrahydroxy-flavone-3-O-β-D-mannopyranoside (**47**)	Aerial parts	EtOAc fraction of MeOH extract	464	C_21_H_20_O_12_	China	[52]
Quercetin-3-O-sulphate (**48**)	Aerial parts	Acidified MeOH	382	C_15_H_10_O_10_S	Seberang Perai, Malaysia	[55]
Myricetin (**49**)	Aerial parts	Acidified 50% MeOH	318	C_15_H_10_O_8_	Bogor, west Java, Indonesia	[29]
Kaempferol (**50**)	Aerial parts	Acidified 50% MeOH	286	C_15_H_10_O_6_	Bogor, west Java, Indonesia	[29]
6-Hydroxykaempferol 7-glucoside (**51**)			464	C_20_H_20_O_12_	Hat Yai, Songkhla, Thailand	[45]
4′,5,7-Trihydroxyflavone-3-O-β-D-glucoside (**52**)	Aerial parts	EtOAc fraction of MeOH extract	448	C_21_H_20_O_11_	China	[52]
Apigenin (**53**)	Aerial parts	Acidified 50% MeOH	270	C_15_H_10_O_5_	Bogor, west Java, Indonesia	[29]
	Aerial parts	EtOAc fraction of MeOH extract	-	-	Chantaburi, Thailand	[59]
Apigenin 7-(2″,3″diacetylglucoside (**54**)						[45]
Chrysoeriol (**55**)	Aerial parts	EtOAc fraction of MeOH extract	300	C_16_H_12_O_6_	Chantaburi, Thailand	[59]
Luteolin (**56**)	Aerial parts	Acidified 50% MeOH	286	C_15_H_10_O_6_	Bogor, west Java, Indonesia	[29]
	Aerial parts	EtOAc fraction of MeOH extract	-	-	Chantaburi, Thailand	[59]
8-Hydroxyluteolin 8-glucoside (**57**)	Leaves	EtOH extract	464	C_20_H_20_O_12_	Hat Yai, Songkhla, Thailand	[45]
Phenolic acids, aldehydes, esters, and ketones						
P-Hydroxybenzoic acid (**58**)	Aerial parts	CHCl_3_ fraction of EtOH extract	138	C_7_H_6_O_3_	Hepu, Guangxi, China	[51]
3,4,5-Trimethoxybenzoic acid (**59**)	Roots	CH_2_Cl_2_ fraction of MeOH extract	212	C_10_H_12_O_5_	Kaohsiung, Taiwan	[50]
Caffeic acid (**60**)	Aerial parts	EtOAc fraction of MeOH extract	180	C_9_H_8_O_4_	China	[52]
P-Hydroxybenzaldehyde (**61**)	Roots	CH_2_Cl_2_ fraction of MeOH extract	122	C_7_H_6_O_2_	Kaohsiung, Taiwan	[50]
3,4-Dihydroxy benzaldehyde (**62**)	Aerial parts	CHCl_3_ fraction of EtOH extract	138	C_7_H_6_O_3_	Hepu, Guangxi, China	[51]
Vanillin (**63**)	Aerial parts	CHCl_3_ fraction of EtOH extract	152	C_8_H_8_O_3_	Hepu, Guangxi, China	[51]
3,4-Dihydroxy-5-methoxybenzaldehyde (**64**)	Aerial parts	EtOAc fraction of MeOH extract	168	C_8_H_8_O_4_	China	[52]
	Aerial parts	CHCl_3_ fraction of EtOH extract	-	-	Hepu, Guangxi, China	[51]
Syringicaldehyde (**65**)	Aerial parts	CHCl_3_ fraction of EtOH extract	182	C_9_H_10_O_4_	Hepu, Guangxi, China	[51]
Trans-Coniferyl aldehyde (**66**)	Aerial parts	CHCl_3_ fraction of EtOH extract	178	C_10_H_10_O_3_	Hepu, Guangxi, China	[51]
Dibutylphthalate (**67**)	Aerial parts	CHCl_3_ fraction of EtOH extract	278	C_16_H_22_O_4_	Hepu, Guangxi, China	[51]
Ethyl caffeate (**68**)	Aerial parts	CHCl_3_ fraction of EtOH extract	208	C_11_H_12_O_4_	Hepu, Guangxi, China	[51]
2,3-Dihydroxy-1-(4-hydroxy-3-methoxyphenyl)-propan-1-one (**69**)	Aerial parts	CHCl_3_ fraction of EtOH extract	212	C_10_H_12_O_5_	Hepu, Guangxi, China	[51]
Phenolics and phenolic glucosides						
1,2-Bis-(4-Hydroxy-3-methoxyphenyl)-propane-1,3-diol (erythro) (**70**)	Aerial parts	Polar fraction of MeOH extract	320	C_17_H_20_O_6_	Drigh Road, Karachi, Pakistan	[47]
1,2-Bis-(4-Hydroxy-3-methoxyphenyl)-propane-1,3-diol (threo) (**71**)	Aerial parts	Polar fraction of MeOH extract	320	C_17_H_20_O_6_	Drigh Road, Karachi, Pakistan	[47]
1-(4-Hydroxy-3-methoxyphenyl)-2-{4-[(1E)-3-hydroxyprop-1-en-1-yl]-2-methoxyphenoxy}-propane-1,3-diol (erythro) (**72**)	Aerial parts	Polar fraction of MeOH extract	360	C_20_H_24_O_6_	Drigh Road, Karachi, Pakistan	[47]
1-(4-Hydroxy-3-methoxyphenyl)-2-{4-[(1E)-3-hydroxyprop-1-en-1-yl]-2-methoxyphenoxy}-propane-1,3-diol (threo) (**73**)	Aerial parts	Polar fraction of MeOH extract	360	C_20_H_24_O_6_	Drigh Road, Karachi, Pakistan	[47]
Esculetin (**74**)	Aerial parts	CHCl_3_ fraction of EtOH extract	178	C_9_H_6_O_4_	Hepu, Guangxi, China	[51]
3,4,5-Trimethoxyphenyl-β-D-glucopyranoside (**75**)	Roots	CH_2_Cl_2_ fraction of MeOH extract	346	C_15_H_22_O_9_	Kaohsiung, Taiwan	[50]
Benzyl glucopyranoside (**76**)	Aerial parts	Polar fraction of MeOH extract	270	C_13_H_18_O_6_	Drigh Road, Karachi, Pakistan	[47]
Phenylethyl glucopyranoside (**77**)	Aerial parts	Polar fraction of MeOH extract	284	C_14_H_20_O_6_	Drigh Road, Karachi, Pakistan	[47]
Methyl salicylate glucoside (**78**)	Aerial parts	Polar fraction of MeOH extract	314	C_14_H_18_O_8_	Drigh Road, Karachi, Pakistan	[47]
4-Allyl-2-methoxy-6-hydroxyphenylglucoside (**79**)	Aerial parts	EtOAc fraction of MeOH extract	342	C_16_H_22_O_8_	China	[52]
4-Allyl-2,6-dimethoxy phenyl glucopyranoside (**80**)	Aerial parts	Polar fraction of MeOH extract	356	C_17_H_24_O_8_	Drigh Road, Karachi, Pakistan	[47]
Eugenyl glucoside (Citrucin C) (**81**)	Aerial parts	Polar fraction of MeOH extract	326	C_16_H_22_O_7_	Drigh Road, Karachi, Pakistan	[47]
Tangshenoside Ⅱ (**82**)	Aerial parts	EtOAc fraction of MeOH extract	372	C_17_H_24_O_9_	China	[52]
Thalictoside (**83**)	Roots	CH_2_Cl_2_ fraction of MeOH extract	329	C_14_H_19_NO_8_	Kaohsiung, Taiwan	[50]
Lignans and their derivatives						
(+)-Syringaresinol (**84**)	Roots	CH_2_Cl_2_ fraction of MeOH extract	418	C_22_H_26_O_8_	Kaohsiung, Taiwan	[50]
(+)-Diasyringaresinol (**85**)	Roots	CH_2_Cl_2_ fraction of MeOH extract	418	C_22_H_26_O_8_	Kaohsiung, Taiwan	[50]
	Aerial parts	CHCl_3_fraction of EtOH extract	-	-	Hepu, Guangxi, China	[51]
(+)-Epi-Syringaresinol (**86**)	Roots	CH_2_Cl_2_ fraction of MeOH extract	418	C_22_H_26_O_8_	Kaohsiung, Taiwan	[50]
Syringaresinol monoglucopyranoside (**87**)	Aerial parts	Polar fraction of MeOH extract	580	C_28_H_36_O_13_	Drigh Road, Karachi, Pakistan	[47]
Pinoresinol (**88**)	Aerial parts	EtOAc fraction of MeOH extract	358	C_20_H_22_O_6_	China	[52]
(+)-Epi-Pinoresinol (**89**)	Aerial parts	EtOAc fraction of MeOH extract	358	C_20_H_22_O_6_	China	[52]
Pinoresinol monoglucopyranoside (**90**)	Aerial parts	Polar fraction of MeOH extract	520	C_26_H_32_O_11_	Drigh Road, Karachi, Pakistan	[47]
Liriodendrin (**91**)	Roots	CH_2_Cl_2_ fraction of MeOH extract	742	C_34_H_46_O_18_	Kaohsiung, Taiwan	[50]
Plucheoside D_1_ (**92**)	Aerial parts	Polar fraction of MeOH extract	518	C_26_H_30_O_11_	Drigh Road, Karachi, Pakistan	[48]
Plucheoside D_2_ (**93**)	Aerial parts	Polar fraction of MeOH extract	548	C_27_H_32_O_12_	Drigh Road, Karachi, Pakistan	[48]
Plucheoside D_3_ (**94**)	Aerial parts	Polar fraction of MeOH extract	534	C_27_H_34_O_11_	Drigh Road, Karachi, Pakistan	[48]
Hedyotisol A (**95**)	Aerial parts	Polar fraction of MeOH extract	810	C_42_H_50_O_16_	Drigh Road, Karachi, Pakistan	[47]
Hedyotisol B (**96**)	Aerial parts	Polar fraction of MeOH extract	810	C_42_H_50_O_16_	Drigh Road, Karachi, Pakistan	[47]
Trans-Trismethoxy resveratrol-d4 (**97**)	Leaves	EtOH extract	274	C_17_H_14_D_4_O_3_	Hat Yai, Songkhla, Thailand	[45]
Threo-2,3-Bis(4-hydroxy-3-methoxyphenyl)-3-ethoxypropan-1-ol (**98**)	Aerial parts	EtOAc fraction of EtOH extract	348	C_19_H_24_O_6_	Hepu, Guangxi, China	[51]
Erythro-2,3-Bis(4-hydroxy-3-methoxyphenyl)-3-ethoxypropan-1-ol (**99**)	Aerial parts	EtOAc fraction of EtOH extract	348	C_19_H_24_O_6_	Hepu, Guangxi, China	[51]
(+)-Isolariciresinol (**100**)	Aerial parts	EtOAc fraction of EtOH extract	360	C_20_H_24_O_6_	Hepu, Guangxi, China	[51]
(+)-9′-Isovaleryllariciresinol (**101**)	Aerial parts	EtOAc fraction of EtOH extract	444	C_25_H_32_O_7_	Hepu, Guangxi, China	[51]
Thiophenes and their derivatives						
2-(Pro-1-ynyl)-5-(5,6-dihydroxypenta-1,3-diynyl) thiophene (PYDDT) = 2-(Prop-1-ynyl)-5(5,6-dihydroxyhexa-1, 3-diynyl)-thiophene = PITC-2 = R/J/3 (**102**)	Roots	EtOAc fraction of MeOH extract	230	C_13_H_10_O_2_S	West Bengal, India	[60]
	Aerial parts	*n*-Hexane fraction of MeOH extract	-	-	Chantaburi, Thailand	[59]
	Leaves	*n*-Hexane fraction of MeOH extract	-	-	Gia Lam, Hanoi, Vietnam	[53]
	Roots	EtOAc fraction of MeOH extract	-	-	West Bengal, India	[61]
	Twigs	*n*-Hexane fraction of MeOH extract	-	-	Vietnam	[54]
2-(4-Hydroxy-3-methoxybut-1-yn-1-yl)-5-(penta-1,3-diyn-1-yl)thiophene (**103**)	Aerial parts	EtOAc fraction of MeOH extract	244	C_14_H_12_O_2_S	Guangzhou, China	[62]
2-(3,4-Dihydroxybut-1-yn-1-yl)-5-(penta-1,3-diyn1-yl)thiophene (**104**)	Aerial parts	EtOAc fraction of MeOH extract	230	C_13_H_10_O_2_S	Guangzhou, China	[62]
2-(3-Acetoxy-4-hydroxybut-1-yn-1-yl)-5-(penta-1,3-diyn-1-y1) thiophene (**105**)	Aerial parts	EtOAc fraction of MeOH extract	272	C_15_H_12_O_3_S	Guangzhou, China	[62]
2-(Prop-1-yn-1-yl)-5-(6-acetoxy-5-hydroxyhexa-1,3-diyn-1-yl) thiophene = 2-(Prop-1-inyl)-5-(6-acetoxy-5-hydroxyhexa-1,3-diinyl) thiophene (**106**)	Aerial parts	EtOAc fraction of MeOH extract	272	C_15_H_12_O_3_S	Guangzhou, China	[62]
	Aerial parts	*n*-Hexane fraction of MeOH extract	-	-	Chantaburi, Thailand	[59]
2-(Penta-1,3-diyn-1-yl)-5-(4-acetoxy-3-hydroxybuta-1-yn-1-yl) thiophene (**107**)	Aerial parts	*n*-Hexane fraction of MeOH extract	272	C_15_H_12_O_3_S	Chantaburi, Thailand	[59]
3″R-Pluthiophenol (**108**)	Aerial parts	*n*-Hexane fraction of EtOH extract	230	C_13_H_10_O_2_S	Hepu, Guangxi, China	[51]
3″R-Pluthiophenol-4″-acetate (**109**)	Aerial parts	*n*-Hexane fraction of EtOH extract	272	C_15_H_12_O_3_S	Hepu, Guangxi, China	[51]
3″-Ethoxy-3″S-pluthiophenol (**110**)	Aerial parts	*n*-Hexane fraction of EtOH extract	258	C_15_H_14_O_2_S	Hepu, Guangxi, China	[51]
3″-Ethoxy-3″S-pluthiophenol-4″-acetate (**111**)	Aerial parts	*n*-Hexane fraction of EtOH extract	300	C_17_H_16_O_3_S	Hepu, Guangxi, China	[51]
2-(4-O-β-Glucopyranosyl-3-hydroxybut-1-yn-1-yl)-5-(penta-1,3-diyn-1-yl)thiophene (**112**)	Aerial parts	EtOAc fraction of MeOH extract	392	C_19_H_20_O_7_S	Guangzhou, China	[62]
Other metabolites						
1-Dotriacontanol (**113**)	Leaves	*n*-Hexane fraction of MeOH extract	466	C_32_H_66_O	Gia Lam, Hanoi, Vietnam	[53]
9,12,13-Trihydroxyoctadeca-10(E),15(Z)-dienoic acid (**114**)	Aerial parts	*n*-Hexane fraction of EtOH extract	328	C_18_H_32_O_5_	Hepu, Guangxi, China	[51]
Pinellic acid (**115**)	Aerial parts	*n*-Hexane fraction of EtOH extract	330	C_18_H_34_O_5_	Hepu, Guangxi, China	[51]
1-Eicosanoyl glycerol (**116**)	Twigs	*n*-Hexane fraction of MeOH extract	386	C_23_H_46_O_4_	Vietnam	[54]
Methyl 9-hydroxynonanoate (**117**)	Aerial parts	*n*-Hexane fraction of EtOH extract	188	C_10_H_20_O_3_	Hepu, Guangxi, China	[51]
Triethyl citrate (**118**)	Aerial parts	*n*-Hexane fraction of EtOH extract	276	C_12_H_20_O_7_	Hepu, Guangxi, China	[51]
(Z)-3-Hexenyl glucopyranoside (**119**)	Aerial parts	Polar fraction of MeOH extract	262	C_12_H_22_O_6_	Drigh Road, Karachi, Pakistan	[47]
Fraxinellone (**120**)	Aerial parts	*n*-Hexane fraction of EtOH extract	232	C_14_H_16_O_3_	Hepu, Guangxi, China	[51]
Adenosine (**121**)	Aerial parts	EtOAc fraction of EtOH extract	267	C_10_H_13_N_5_O_4_	Hepu, Guangxi, China	[51]
Uridine 5′-monophosphate (**122**)	Aerial parts	EtOAc fraction of MeOH extract	324	C_9_H_13_N_2_O_9_P	China	[52]

**Table 3:** Biological activity of reported metabolites from *Pluchea indica*.

Compound Name	Biological Activity	Assay, Organism, or Cell Line	Biological Results		Ref.
			Compound	Positive Control	
Caryolane-1,9β-diol (**20**)	Anti-inflammatory/inhibition of NO production	LPS-stimulated production in RAW 264.7 macrophages cells	104.8 (NRC % inhibition)	Dexamethasone 62.2 (NRC % inhibition)	[51]
(8R,9R)-Isocaryolane-8,9-diol (**21**)	Anti-inflammatory/inhibition of NO production	LPS-stimulated production in RAW 264.7 macrophages cells	95.1 (NRC % inhibition)	Dexamethasone 62.2 (NRC % inhibition)	[51]
Clovane-2α,9β-diol (**22**)	Anti-inflammatory/inhibition of NO production	LPS-stimulated production in RAW 264.7 macrophages cells	101.6 (NRC % inhibition)	Dexamethasone 62.2 (NRC % inhibition)	[51]
Valenc-1(10)-ene-8,11-diol (**23**)	Anti-inflammatory/inhibition of NO production	LPS-stimulated production in RAW 264.7 macrophages cells	103.8 (NRC % inhibition)	Dexamethasone 62.2 (NRC % inhibition)	[51]
Stigmasterol (**29**)	Anti-inflammatory/inhibition of NO production	LPS-stimulated production in RAW 264.7 macrophages cells	92.5 (NRC % inhibition)	Dexamethasone 62.2 (NRC % inhibition)	[51]
3,5-Di-O-caffeoylquinic acid (**38**)	α-Glucosidase inhibition	Colorimetric/Rat intestinal maltase	1166 µM (IC_50_)	Acarbose 0.5 µM (IC_50_)	[57]
4,5-Di-O-caffeoylquinic acid methyl ester (**40**)	α-Glucosidase inhibition	Colorimetric/Rat intestinal maltase	208.0 µM (IC_50_)	Acarbose 0.5 µM (IC_50_)	[57]
3,4,5-Tri-O-caffeoylquinic acid (**41**)	α-Glucosidase inhibition	Colorimetric/Rat intestinal maltase	13.0 µM (IC_50_)	Acarbose 0.5 µM (IC_50_)	[57]
	Collagenase inhibition	Fluorometric/Collagenase type IV	1.5 µM (IC_50_)	Phosphramidon 7.4 µM (IC_50_)	[58]
	MMP-2 inhibition	Fluorometric/MMP-2 proenzyme	2.5 µM (IC_50_)	Chlorhexidine 7.3 µM (IC_50_)	[58]
	MMP-9 inhibition	Fluorometric/MMP-9 monomer	6.4 µM (IC_50_)	Chlorhexidine 25.2 µM (IC_50_)	[58]
3,4,5-tri-O-caffeoylquinic acid methyl ester (**42**)	α-Glucosidase inhibition	Colorimetric/Rat intestinal maltase	2.0 µM (IC_50_)	Acarbose 0.5 µM (IC_50_)	[57]
1,3,4,5-Tetra-O-Caffeoylquinic acid (**43**)	α-Glucosidase inhibition	Colorimetric/Rat intestinal maltase	11.0 µM (IC_50_)	Acarbose 0.5 µM (IC_50_)	[57]
	Collagenase inhibition	Fluorometric/Collagenase type IV	6.3 µM (IC_50_)	Phosphramidon 7.4 µM (IC_50_)	[58]
	MMP-2 inhibition	Fluorometric/MMP-2 proenzyme	18.4 µM (IC_50_)	Chlorhexidine 7.3 µM (IC_50_)	[58]
	MMP-9 inhibition	Fluorometric/MMP-9 monomer	16.8 µM (IC_50_)	Chlorhexidine 25.2 µM (IC_50_)	[58]
Quercetin (**44**)	Collagenase inhibition	Fluorometric/Collagenase type IV	16.9 µM (IC_50_)	Phosphramidon 7.4 µM (IC_50_)	[58]
	CYP2A6 inhibition	Enzymatic reconstitution	2.66 µM (IC_50_)	Methoxsalen 0.19 µM (IC_50_)	[59]
	CYP2A13 inhibition		0.80 µM (IC_50_)	Methoxsalen 0.43 µM (IC_50_)	[59]
Apigenin (**53**)	CYP2A6 inhibition	Enzymatic reconstitution	0.9 µM (IC_50_)	Methoxsalen 0.19 µM (IC_50_)	[59]
	CYP2A13 inhibition		0.05 µM (IC_50_)	Methoxsalen 0.43 µM (IC_50_)	[59]
Luteolin (**56**)	CYP2A6 inhibition	Enzymatic reconstitution	1.38 µM (IC_50_)	Methoxsalen 0.19 µM (IC_50_)	[59]
	CYP2A13 inhibition		0.18 µM (IC_50_)	Methoxsalen 0.43 µM (IC_50_)	[59]
Chrysoeriol (**55**)	CYP2A6 inhibition	Enzymatic reconstitution	1.14 µM (IC_50_)	Methoxsalen 0.19 µM (IC_50_)	[59]
	CYP2A13 inhibition		0.82 µM (IC_50_)	Methoxsalen 0.43 µM (IC_50_)	[59]
3,4-Dihydroxy benzaldehyde (**62**)	Anti-inflammatory/inhibition of NO production	LPS-stimulated production in RAW 264.7 macrophages cells	92.9 (NRC % inhibition)	Dexamethasone 62.2 (NRC % inhibition)	[51]
Vanillin (**63**)	Anti-inflammatory/inhibition of NO production	LPS-stimulated production in RAW 264.7 macrophages cells	99.6 (NRC % inhibition)	Dexamethasone 62.2 (NRC % inhibition)	[51]
3,4-Dihydroxy-5-methoxybenzaldehyde (**64**)	Anti-inflammatory/inhibition of NO production	LPS-stimulated production in RAW 264.7 macrophages cells	103.9 (NRC % inhibition)	Dexamethasone 62.2 (NRC % inhibition)	[51]
Syringicaldehyde (**65**)	Anti-inflammatory/inhibition of NO production	LPS-stimulated production in RAW 264.7 macrophages cells	92.6 (NRC % inhibition)	Dexamethasone 62.2 (NRC % inhibition)	[51]
Trans-Coniferyl aldehyde (**66**)	Anti-inflammatory/inhibition of NO production	LPS-stimulated production in RAW 264.7 macrophages cells	94.2 (NRC % inhibition)	Dexamethasone 62.2 (NRC % inhibition)	[51]
Dibutylphthalate (**67**)	Anti-inflammatory/inhibition of NO production	LPS-stimulated production in RAW 264.7 macrophages cells	101.1 (NRC % inhibition)	Dexamethasone 62.2 (NRC % inhibition)	[51]
Ethyl caffeate (**68**)	Anti-inflammatory/inhibition of NO production	LPS-stimulated production in RAW 264.7 macrophages cells	77.9 (NRC % inhibition)	Dexamethasone 62.2 (NRC % inhibition)	[51]
2,3-Dihydroxy-1-(4-hydroxy-3-methoxyphenyl)-propan-1-one (**69**)	Anti-inflammatory/inhibition of NO production	LPS-stimulated production in RAW 264.7 macrophages cells	100.9 (NRC % inhibition)	Dexamethasone 62.2 (NRC % inhibition)	[51]
Esculetin (**74**)	Anti-inflammatory/inhibition of NO production	LPS-stimulated production in RAW 264.7 macrophages cells	88.5 (NRC % inhibition)	Dexamethasone 62.2 (NRC % inhibition)	[51]
(+)-Diasyringaresinol (**85**)	Anti-inflammatory/inhibition of NO production	LPS-stimulated production in RAW 264.7 macrophages cells	101.7 (NRC % inhibition)	Dexamethasone 62.2 (NRC % inhibition)	[51]
Threo-2,3-Bis(4-hydroxy-3-methoxyphenyl)-3-ethoxypropan-1-ol (**98**)	Anti-inflammatory/inhibition of NO production	LPS-stimulated production in RAW 264.7 macrophages cells	101.7 (NRC % inhibition)	Dexamethasone 62.2 (NRC % inhibition)	[51]
Erythro-2,3-Bis(4-hydroxy-3-methoxyphenyl)-3-ethoxypropan-1-ol (**99**)	Anti-inflammatory/inhibition of NO production	LPS-stimulated production in RAW 264.7 macrophages cells	99.7 (NRC % inhibition)	Dexamethasone 62.2 (NRC % inhibition)	[51]
(+)-Isolariciresinol (**100**)	Anti-inflammatory/inhibition of NO production	LPS-stimulated production in RAW 264.7 macrophages cells	101.9 (NRC % inhibition)	Dexamethasone 62.2 (NRC % inhibition)	[51]
(+)-9′-Isovaleryllariciresinol (**101**)	Anti-inflammatory/inhibition of NO production	LPS-stimulated production in RAW 264.7 macrophages cells	77.6 (NRC % inhibition)	Dexamethasone 62.2 (NRC % inhibition)	[51]
2-(Prop-1-inyl)-5-(5,6-dihydroxyhexa-1,3-diinyl) thiophene (**102**)	CYP2A6 inhibition	Enzymatic reconstitution	3.90 µM (IC_50_)	Methoxsalen 0.19 µM (IC_50_)	[59]
	CYP2A13 inhibition		2.40 µM (IC_50_)	Methoxsalen 0.43 µM (IC_50_)	[59]
	Anti-amoebic	Cell count/*Entamoeba histolytica* (HM1)	50 µg/mL (MIC)	Metronidazole 5 µg/mL (MIC)	[60]
2-(Prop-1-inyl)-5-(6-acetoxy-5-hydroxyhexa-1, 3-diinyl) thiophene (**106**)	CYP2A6 inhibition	Enzymatic reconstitution	4.44 µM (IC_50_)	Methoxsalen 0.19 µM (IC_50_)	[59]
	CYP2A13 inhibition		2.94 µM (IC_50_)	Methoxsalen 0.43 µM (IC_50_)	[59]
2-(Penta-1,3-diyn-1-yl)-5-(4-acetoxy-3-hydroxybuta-1-yn-1-yl) thiophene (**107**)	CYP2A6 inhibition	Enzymatic reconstitution	6.43 µM (IC_50_)	Methoxsalen 0.19 µM (IC_50_)	[59]
	CYP2A13 inhibition		6.18 µM (IC_50_)	Methoxsalen 0.43 µM (IC_50_)	[59]
3″R-Pluthiophenol (**108**)	Anti-inflammatory/inhibition of NO production	LPS-stimulated production in RAW 264.7 macrophages cells	84.5 (NRC % inhibition)	Dexamethasone 62.2 (NRC % inhibition)	[51]
3″R-Pluthiophenol-4″-acetate (**109**)	Anti-inflammatory/inhibition of NO production	LPS-stimulated production in RAW 264.7 macrophages cells	83.4 (NRC % inhibition)	Dexamethasone 62.2 (NRC % inhibition)	[51]
3″-Ethoxy-3″S-pluthiophenol (**110**)	Anti-inflammatory/inhibition of NO production	LPS-stimulated production in RAW 264.7 macrophages cells	86.9 (NRC % inhibition)	Dexamethasone 62.2 (NRC % inhibition)	[51]
3″-Ethoxy-3″S-pluthiophenol-4″-acetate (**111**)	Anti-inflammatory/inhibition of NO production	LPS-stimulated production in RAW 264.7 macrophages cells	90.1 (NRC % inhibition)	Dexamethasone 62.2 (NRC % inhibition)	[51]
9,12,13-Trihydroxyoctadeca-10(E),15(Z)-dienoic acid (**114**)	Anti-inflammatory/inhibition of NO production	LPS-stimulated production in RAW 264.7 macrophages cells	90.3 (NRC % inhibition)	Dexamethasone 62.2 (NRC % inhibition)	[51]
Pinellic acid (**115**)	Anti-inflammatory/inhibition of NO production	LPS-stimulated production in RAW 264.7 macrophages cells	89.5 (NRC % inhibition)	Dexamethasone 62.2 (NRC % inhibition)	[51]
Methyl 9-hydroxynonanoate (**117**)	Anti-inflammatory/inhibition of NO production	LPS-stimulated production in RAW 264.7 macrophages cells	93.6 (NRC % inhibition)	Dexamethasone 62.2 (NRC % inhibition)	[51]
Triethyl citrate (**118**)	Anti-inflammatory/inhibition of NO production	LPS-stimulated production in RAW 264.7 macrophages cells	91.1 (NRC % inhibition)	Dexamethasone 62.2 (NRC % inhibition)	[51]
Fraxinellone (**120**)	Anti-inflammatory/inhibition of NO production	LPS-stimulated production in RAW 264.7 macrophages cells	52.1 (NRC % inhibition)	Dexamethasone 62.2 (NRC % inhibition)	[51]
Adenosine (**121**)	Anti-inflammatory/inhibition of NO production	LPS-stimulated production in RAW 264.7 macrophages cells	88.7 (NRC % inhibition)	Dexamethasone 62.2 (NRC % inhibition)	[51]

## Data Availability

Not applicable.

## Abbreviations

AA: Arachidonic acid; AAPH: 2,2′-azobis(2-amidinopropane) dihydrochloride; ABTS: 2,2′-Azino-bis-(3-ethylbenzthiazoline-6-sulphonic acid; ADP: Adenosine di-phosphate; ALP: Alkaline phosphatase; ALT: Alanine transaminase; AVO: Acidic vesicular organelle; Bcl-2: β-Cell lymphoma 2; BUN: Blood urea nitrogen; CBC: Complete blood count; CH2Cl2: Dichloromethane; CHCl3: Chloroform; DPPH: 2,2-Diphenyl-1-picrylhydrazyl; EA.hy926: Human vascular endothelial cells; EAC: Ehrlich ascites carcinoma; CYP 2A13: Cytochrome P450 2A13; CYP 2A6: Cytochrome P450 2A6; EC50: Effective Concentration at 50% of the total effect; EPP: Ethylphenylpropiolate; EV: Edema volume; EtOAc: Ethyl acetate; EtOH: Ethanol; FRAP: Ferric Antioxidant Power; GABA: gamma-Aminobutyric acid GAE/g: Gallic acid equivalent/g: GBM: Human glioblastoma cells; GBM8401: Human glioma cells; GC-MS: Gas chromatography–mass spectrometry; HDL-C: High-density lipoprotein cholesterol; HO-1: Heme oxygenase-1; HFD: High fat diet; HeLa: Cervical carcinoma cells; HOCl: Hypochlorous acid; HPLC: High-pressure liquid chromatography; IC50: concentration of inhibitor at 50% of the total inhibition; ; ICAM-1: Intercellular adhesion molecule-1; IFN-γ: Interferon-γ; IL-1β: Interlukin1-β; iNOS: Inducible nitric oxide synthase; Ki-67: Nuclear protein; LC3-II: Microtubule-associated light chain 3-II; LC-MC: Liquid chromatography–mass spectrometry; LDL: Low-density lipoproteins; LDL-C: Low-density lipoprotein cholesterol; LPS: Lipopolysaccharide; MeOH: Methanol; MIC: The minimum inhibitory concentration; MMP-2: Matrix metalloproteinase-2; MMP-9: Matrix metalloproteinase-9; MTT: 3-(4,5-Dimethylthiazol-2-yl)-2,5-diphenyl tetrazolium bromide; n-BuOH: NADPH: Nicotinamide adenine dinucleotide phosphate; NMR: Nuclear magnetic resonance; NF-κBp65: Nuclear factor-κBp65; NO: Nitrous oxide; NRC: Nitrite relative concentration; OC: Orthogonal chromatography: PAF: Platelet activation factor; PGE2: Prostaglandin E2; PKC: Protein kinase C; PITC 2: 2-(Prop-1-ynyl)-5(5,6-dihydroxyhexa-1,3-diynyl)-thiophene; pSTAT1: Signal transducer and activator of transcription 1; ROS: Reactive oxygen species; RP-18: Reversed phase C18 silica gel; SIN-1: 3-Morpholinosydnonimine hydrochloride; pSTAT1: Signal transducer and activator of transcription 1; SiO2 CC: Silica gel column chromatography; SLN: Solid lipid nanoparticle; STZ: Streptozotocin; TBA: 2-Thiobarbituric acid; TBARS: Thiobarbituric Acid Reactive Substances; TE/g: Total extract/gram; TEAC: Trolox equivalent antioxidant capacity; TFC: Total flavonoid contents; TG: Triglyceride; TNF-α: Tumor necrosis factor-α; VCAM-1: Vascular cell adhesion molecule-1; WOF: Warmed-over flavor.
